# Color and contrast vision in mouse models of aging and Alzheimer’s disease using a novel visual-stimuli four-arm maze

**DOI:** 10.1038/s41598-021-80988-0

**Published:** 2021-01-13

**Authors:** Jean-Philippe Vit, Dieu-Trang Fuchs, Ariel Angel, Aharon Levy, Itschak Lamensdorf, Keith L. Black, Yosef Koronyo, Maya Koronyo-Hamaoui

**Affiliations:** 1 Department of Biomedical Sciences, Cedars-Sinai Medical Center, 127 S. San Vicente Blvd., Los Angeles, CA 90048 USA; 2 Biobehavioral Research Core, Cedars-Sinai Medical Center, Los Angeles, CA USA; 3 Department of Neurosurgery, Maxine Dunitz Neurosurgical Research Institute, Cedars-Sinai Medical Center, Los Angeles, CA USA; 4 Pharmaseed Ltd., 9 Hamazmera St., 74047 Ness Ziona, Israel

**Keywords:** Colour vision, Retina, Alzheimer's disease, Cognitive ageing, Hippocampus, Spatial memory

## Abstract

We introduce a novel visual-stimuli four-arm maze (ViS4M) equipped with spectrally- and intensity-controlled LED emitters and dynamic grayscale objects that relies on innate exploratory behavior to assess color and contrast vision in mice. Its application to detect visual impairments during normal aging and over the course of Alzheimer’s disease (AD) is evaluated in wild-type (WT) and transgenic APP_SWE_/PS1_∆E9_ murine models of AD (AD^+^) across an array of irradiance, chromaticity, and contrast conditions. Substantial color and contrast-mode alternation deficits appear in AD^+^ mice at an age when hippocampal-based memory and learning is still intact. Profiling of timespan, entries and transition patterns between the different arms uncovers variable AD-associated impairments in contrast sensitivity and color discrimination, reminiscent of tritanomalous defects documented in AD patients. Transition deficits are found in aged WT mice in the absence of alternation decline. Overall, ViS4M is a versatile, controlled device to measure color and contrast-related vision in aged and diseased mice.

## Introduction

Mounting evidence indicates that Alzheimer’s Disease (AD) affects the neurosensory retina, a CNS tissue and developmental outgrowth of the diencephalon^1–8^. The pathology of AD in the retina appears to mirror the disease in the brain^9–16^. Specifically, the retina of AD patients exhibits neuronal and pericyte cell loss along with retinal manifestation of AD pathological hallmarks—amyloid β-protein (Aβ) plaques and hyperphosphorylated tau (p-Tau)^9–13,16–27^. Similarly, numerous studies examining the retina of sporadic and transgenic animal models of AD have reported the existence of Aβ deposits and tauopathy that are linked with inflammation and neurodegeneration^9,14,15,28–41^.

The well-documented clinical symptoms of individuals suffering from AD include behavioral changes and progressive cognitive decline^42–44^. Additionally, a growing number of studies also report sleep disturbances as well as various visual impairments and retinal functional abnormalities, as assessed by electroretinogram (ERG) recordings, in patients with mild cognitive impairment (MCI) and AD^11,33,45–61^. Indeed, visual impairments are among the earliest symptoms documented in AD patients, especially loss of contrast sensitivity^54,59,62–65^ and altered color vision reminiscent of tritanomaly, an abnormality of blue-sensitive retinal cones^49,59,65–67^. Both color and contrast sensitivity were found to be significantly impaired in AD patients when compared with healthy individuals^46,53^. Moreover, a recent study found significant correlations between impaired contrast sensitivity and cerebral amyloid and tau burdens among individuals with subjective cognitive decline and MCI^61^.

Since the retina of MCI and AD patients exhibit the pathological hallmarks of AD, vascular abnormalities, and neurodegeneration, these retinal pathologies may explain the functional impairments seen such as loss of contrast and color sensitivity. In fact, studies in various transgenic mouse models of AD suggest that retinal Aβ deposits disrupt retinal structure and may contribute to visual deficits^36,37,68–71^. Particularly, the transgenic mice showed disturbances in the visual system, reduced function of ganglion cells and photoreceptors, and accumulation of retinal and cerebral Aβ. Moreover, reduction in inner retinal thickness was associated with atrophy of the visual cortex^68,69^.

In vertebrates, rod and cone photoreceptors contribute directly to sight, specifically to color and contrast sensitivity. Rod cells are more light-sensitive than cones and can react to a single photon of light, thus contributing to vision under dim illumination conditions. Cones are active at brighter light levels and support color vision; they are grouped into wavelength-sensitive (ws) types: short (S; ws blue), medium (M; ws green), and long (L; ws green, yellow and red), with the S cones further subdivided into S1 (ws near-ultraviolet to blue range) and S2 (ws blue range)^72–74^. While humans are trichromatic in daylight vision and possess three spectral types of cones (S, M, and L), mice are dichromatic with only two cone types (S and M)^75–77^. Mice have UV-sensitive S1-opsin that provides greater sensitivity for light detection^78,79^. The S-opsins have also been shown to be activated by wavelengths in the blue range^80^. The mouse retina displays asymmetric and mixed expression of its two cone opsins across the dorsal–ventral axis of the retina, creating opposing gradients. There is more frequent S-opsin in the ventral retina and abundant M-opsin in the dorsal retina^81–84^. True S-cones, containing only S-opsin and implicated in color discrimination in mice^75,85,86^, are sparse in the dorsal retina, but account for ~ 30% of the cone population in the ventral retina^87^. This suggests that in mice, color discrimination and wavelength-specific luminance contrast sensitivity may differ with retinotopic location^74,88^.

Importantly, mice can detect changes in both color and brightness and further use color to guide their behavior^88,89^. They have a potent photopic contrast sensitivity and can detect low-contrast stimuli with a peak contrast threshold of 2%^90,91^. Van Alphen and colleagues showed that in C57BL/6J mice, females have lower contrast sensitivity than males and claim that contrast sensitivity substantially decreases with age^92^. Although rod and cone numbers and densities do not decrease as mice age, the maximal voltages (Vmax) of cone-based ERGs suggest a substantial age-related decline^93–95^.

In previous studies, color vision and contrast sensitivity have been measured in C57BL/6 mice using behavioral assessments like optomotor response^96,97^, optokinetic reflex^92^, and forced-choice procedure. The latter includes the visual water task^89,98^, psychometric curves in freely moving mice^99^, and the visual-stimulation environment^88^. However, the specific visual changes in contrast and color vision were never previously demonstrated in transgenic mouse models of AD.

Here, we created a novel, high-definition, controlled, and user-friendly behavioral apparatus to detect functional vision changes associated with aging and AD-related pathology in mice. Utilizing this visual-stimuli four-arm maze (ViS4M), we investigated color and contrast sensitivity in three cohorts of WT mice and double-transgenic APP_SWE_/PS1_∆E9_ mouse models of AD (AD^+^ mice). Effects of aging and gender on vision were tested in both strains. Overall, by using an array of mesopic and photopic illuminations in the novel ViS4M, we identified early and progressive impairments in color vision and contrast sensitivity in AD-model mice. Some of these vision changes preceded cognitive decline and predicted cognitive status.

## Results

### A visual-stimuli four-arm maze (ViS4M) to assess spontaneous behavior induced by color

The first goal of this study was to explore color vision changes or impairments that occur in the normal aging mouse and in the double transgenic APP_SWE_/PSEN1_∆E9_ (AD^+^) mouse. It was imperative for us to assess the spontaneous behavior of the mice that would be solely guided by visual stimuli (i.e. irradiance, color). To do so, we constructed a novel behavioral apparatus consisting of four arms of different colors that the mouse can explore freely with no forced choices to make, no introduction of rewards, and importantly, no training. The originality of the ViS4M is that upon entering an arm, the mouse is fully immersed in a colored space with predefined visual stimuli modalities. The frequencies and patterns of exploration of the arms in this new maze can generate a large amount of data related to color, brightness and contrast discrimination and preferences. Different frequencies and patterns in the AD^+^ mouse could be relevant to visual dysfunctions observed in AD patients.

**Figure 1:**
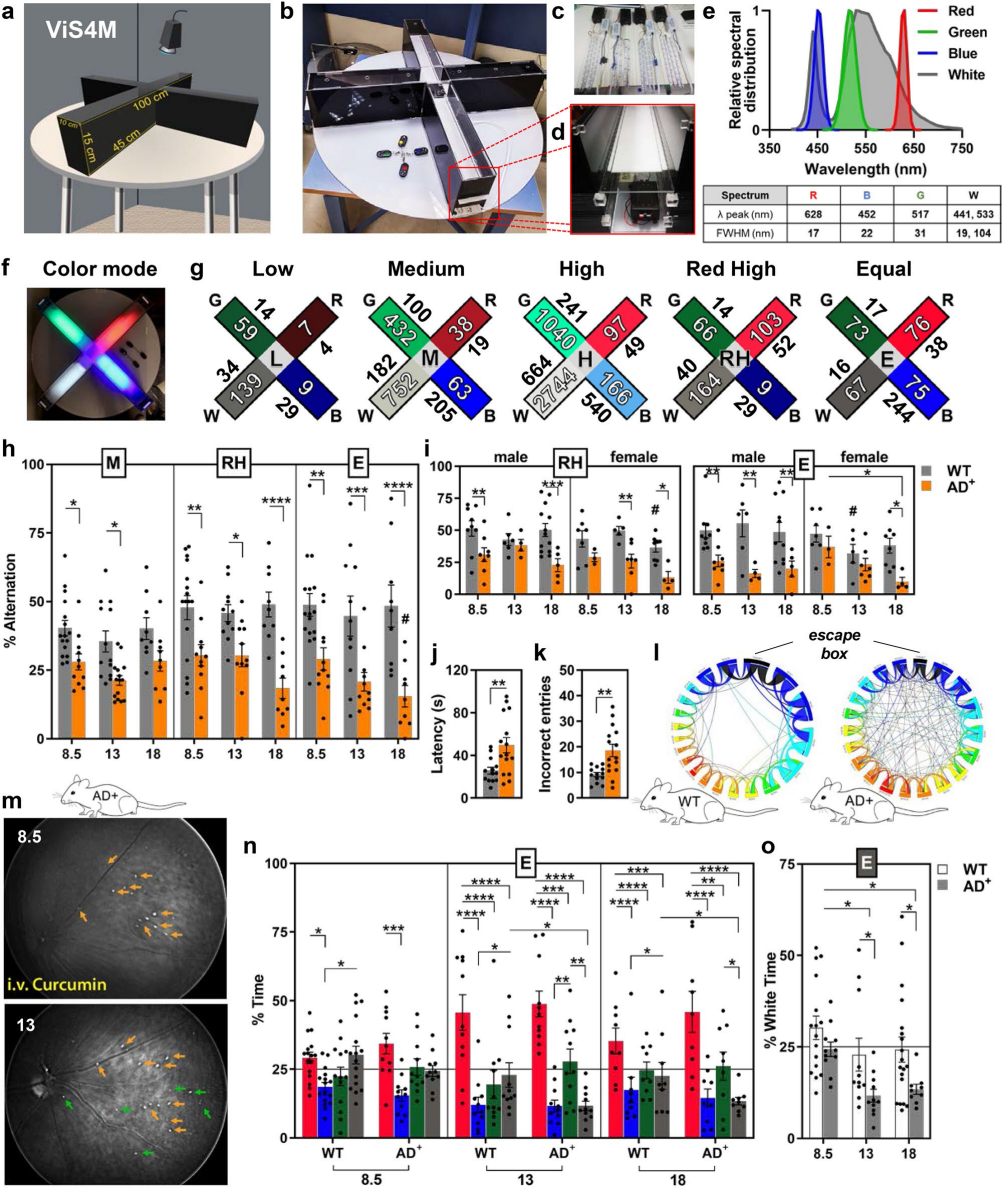
The ViS4M for assessing visual and cognitive function in WT and AD^+^ mice. (**a**–**f**) Description of apparatus and color-based modalities of testing. (**a**) Dimensions of the ViS4M. (**b**–**d**) Accessories and parts: four remote devices (**b**) separately control four LED emitters (**c**), each inserted individually below white removable semi-transparent floors (**d**). (**e**) Relative spectral distribution of red, blue, green and white LED lights and table with peak wavelength(s) and full width at half-maximum (FWHM). (**f**) Each LED emitter provides chromatic light at a single wavelength λ. (**g**) Testing parameters under five light intensity conditions: Low (L), Medium (M), High (H), Red High (RH) and Equal (E), according to illuminance [inside arms] and corresponding irradiance [outside arms] units. Incident illuminance (Lux), specified in each arm, was measured for each color using a Sekonic L-308S light meter (exposure values EV mode and ISO 100); EV were converted to Lux using the relationship *Lux* = *2.5* × *2*^*EV*^. Irradiance (μW/cm^2^) was derived from illuminance (lux) using the freely available Rodent Toolbox V1. (**h**,**i**) Percentage of alternation in 8.5-, 13- and 18-month-old mice under M, RH and E conditions (**h**; # *p* < 0.05 between 8.5 and 18-month-old AD + mice under E condition) and comparison by gender under RH and E conditions (**i**; # *p* < 0.05 between male and female WT at 18 months under RH condition and at the age of 13 months under E condition). Percentage of alternation is defined as the sequential visit of the four different arms without returning to a previously visited arm relative to the total number of sequences of 4 arms (total number of arm entries minus 2). (**j**–**l**) Barnes maze assessment of hippocampus-related spatial memory in the 13-month-old cohort. (**j**) Escape latency in seconds. (**k**) Number of incorrect entries. (**l**) Chord diagrams depicting the search strategy on day 7 (memory retention). (**m**) Non-invasive in vivo retinal imaging of curcumin-positive Aβ plaques in the same AD^+^ mouse at 8.5- and 13-months-old. Orange arrows show plaques present at both time points, while green arrows identify newly formed plaques at 13 months. (**n**) Percentage of time spent in each colored arm of 8.5-, 13- and 18-month-old under E condition. (**o**) Percentage of time spent in the white arm between 8.5-, 13- and 18-month-old under E condition. Mouse cohorts: 8.5-month-old WT (n = 16; 9 males, 7 females) and AD^+^ (n = 11; 8 males, 3 females); 13-month-old WT (n = 11–15; 6–8 males, 5–7 females) and AD^+^ (n = 11–15; 4–7 males, 7–8 females); 18-month-old WT (n = 19; 11 males, 8 females) and AD^+^ (n = 9; 5 males, 4 females). Group means, SEMs and individual data points are shown. * *p* < 0.05, ** *p* < 0.01, *** *p* < 0.001, **** *p* < 0.0001, by two-way ANOVA followed by *posthoc* Fisher’s LSD test.

The four arms of the ViS4M are built with black walls, semi-transparent white floors, and transparent ceilings (Fig. 1a,b). In color mode, illumination-controlled LED emitters are placed below the floors in each arm (Fig. 1b–d). Each LED emitter provides light with a predefined wavelength (Fig. 1e): red [λ peak 628 and full width at half maximum (FWHM) 17 nm], green [λ peak 517 and FWHM 31 nm], blue [λ peak 452 and FWHM 22 nm], and white [λ_1_ 441 and 19 nm; λ_2_ 533 and 104 nm]. The relative sensitivity of the different mouse opsins to these wavelengths is shown (Fig. S1a,b). The criterion for choosing the LED colors is as follows: the red light as a dark-space control arm with low- to no-color stimulus; the green light to stimulate the mouse retinal M-opsin in M-cones, without stimulating S-opsin; and the blue light to stimulate the mouse retinal S-opsin in S- and M-cones, in addition to the M-opsin. The UV lights that can activate solely the S-cones were avoided due to safety considerations for the human researcher and the mouse cornea. The white light is used as a wide-spectrum wavelength stimulus for both mouse S-opsin and M-opsin. Thus, our goal was to create a gradient of S- and M-opsin activation (Fig. S1b), from no stimulation (green and red) to higher stimulation (blue and white) of S-opsin, and from no to low stimulation (red and blue) to high stimulation (green and white) of M-opsin.

The color LED lights traverse the translucent white floors and diffuse in their respective entire arm (Fig. 1f). We tested various levels of predefined illuminations, namely low (L), medium (M), high (H), red high (RH) and equal (E) conditions (Fig. 1g). In all conditions, photometric and radiometric units of light intensity were measured for each stimulus and effective photon flux for each opsin photopigment was estimated by weighting spectral irradiance according to pigment spectral efficiency (Fig S1c).

The response to the different illumination conditions was assessed in three cohorts of 8.5, 13- and 18-month-old AD^+^ mice [total n = 35; 8.5 months-old: n = 11 (30% females), 13 months-old: n = 11–15 (50% females), 18 months-old: n = 9 (40% females)] and age- and gender-matched non-transgenic wild-type littermates [WT; total n = 48; 8.5 months-old: n = 16 (40% females), 13 months-old: n = 11–15 (50% females), 18 months-old: n = 19 (40% females)]. We evaluated different aspects of spontaneous behavior that could indicate arm preference (% entries and % time spent in the colored arms) or discrimination (transition and alternation between arms) that could shed light on visual impairments related to AD pathology and damage to the retina.

### Early and progressive color vision impairments in AD-model mice

Analysis of exploration patterns, as measured by percentage of spontaneous alternation, suggests that WT mice tend to consecutively enter the four different arms of the ViS4M in certain sequences prior to returning to an arm they already visited (Fig. 1h,i; for extended data see Fig. S2a–e). To establish the effect of color stimulus alone in the 8.5-month-old WT mice, we initially measured the percentage of alternation in all intensity conditions as compared to No-Color (NC) condition (Fig. S2d). In these WT mice, we found that alternation under RH and E conditions was significantly higher (46.5% and 49.3%, respectively) than that under NC condition (35.5%) and substantially higher than the 22% chance level probability (Fig. S2e; *p* < 0.05 and *p* < 0.001 respectively). Locomotion in mice was assessed as the total number of entries (Fig. S2f–h) and the distance traveled (Fig. S2i) during each session for all conditions and all age groups. Average speed is also shown for all conditions in the 8.5-month-old mouse cohorts (Fig. S2j). There was an overall increase in activity in AD^+^ mice, especially in the 8.5-month-old cohort (Fig. S2f–j). For the RH and E conditions, no major differences in total entries were observed between males and females in their respective groups (Fig. S2g,h).

Compared to WT, AD^+^ mice showed a dramatic decrease in the percentage of alternation (Fig. 1h; Fig. S2a; 37.9–49.3% in WT vs. 15.5–30.4% in AD^+^ for all conditions and all ages, *p* < 0.05–*p* < 0.0001, by two-way ANOVA and Fisher’s LSD test) that fluctuated around the chance level probability. Importantly, reduced % alternation was observed as early as 8.5 months of age in AD^+^ mice (an early-symptomatic age) in four out of the five conditions of light intensities (Fig. 1h,i; extended data in Fig. S2a–c). While WT mice had a constant % alternation with aging, AD^+^ mice exhibited substantial reductions in % alternation that progressed with aging, only under E condition (Fig. 1h; extended data in Fig. S2a; 29.2% and 15.5% from 8.5- to 18-months of age, respectively, *p* < 0.05), suggesting worsening of visual impairment during disease progression. Analysis of % alternation in females and males separately reveals that both genders of transgenic mice exhibited poorer performances when compared to WT mice (Fig. 1i and Fig. S2b,c). However, the decline in performance relative to gender-matched WT mice appeared consistently earlier in AD^+^ males (8.5 months) than in AD^+^ females. The decline with age was more pronounced for AD^+^ females under the E condition (Fig. 1i).

In comparison, long-term memory was impaired in 13-month-old symptomatic AD^+^ mice on test day 7 of the Barnes maze—a hippocampal-based behavioral test—as shown by higher latencies and incorrect entries (Fig. 1j,k; *p* < 0.01). WT mice rapidly adopted a serial search strategy while AD^+^ mice continued to make a significant number of random incorrect searches in order to find the escape box as shown in chord diagrams (Fig. 1l). These results suggest that the visual stimuli test is more sensitive than the Barnes maze and other behavioral tests for detection of early impairments^100–103^.

To verify the presence and progression of AD pathology in the retina of these mice, we conducted noninvasive retinal curcumin imaging. Representative in vivo images of Aβ plaques in the retina of the same AD^+^ mouse at 8.5 and 13 months of age are shown (Fig. 1m). Retinal Aβ plaques that were detected at 8.5 months (baseline) and persisted at 13 months are indicated by orange arrows, while newly formed plaques (at 13 months) are highlighted by green arrows.

### Reduced preference for white arm in AD-model mice

**Figure 2:**
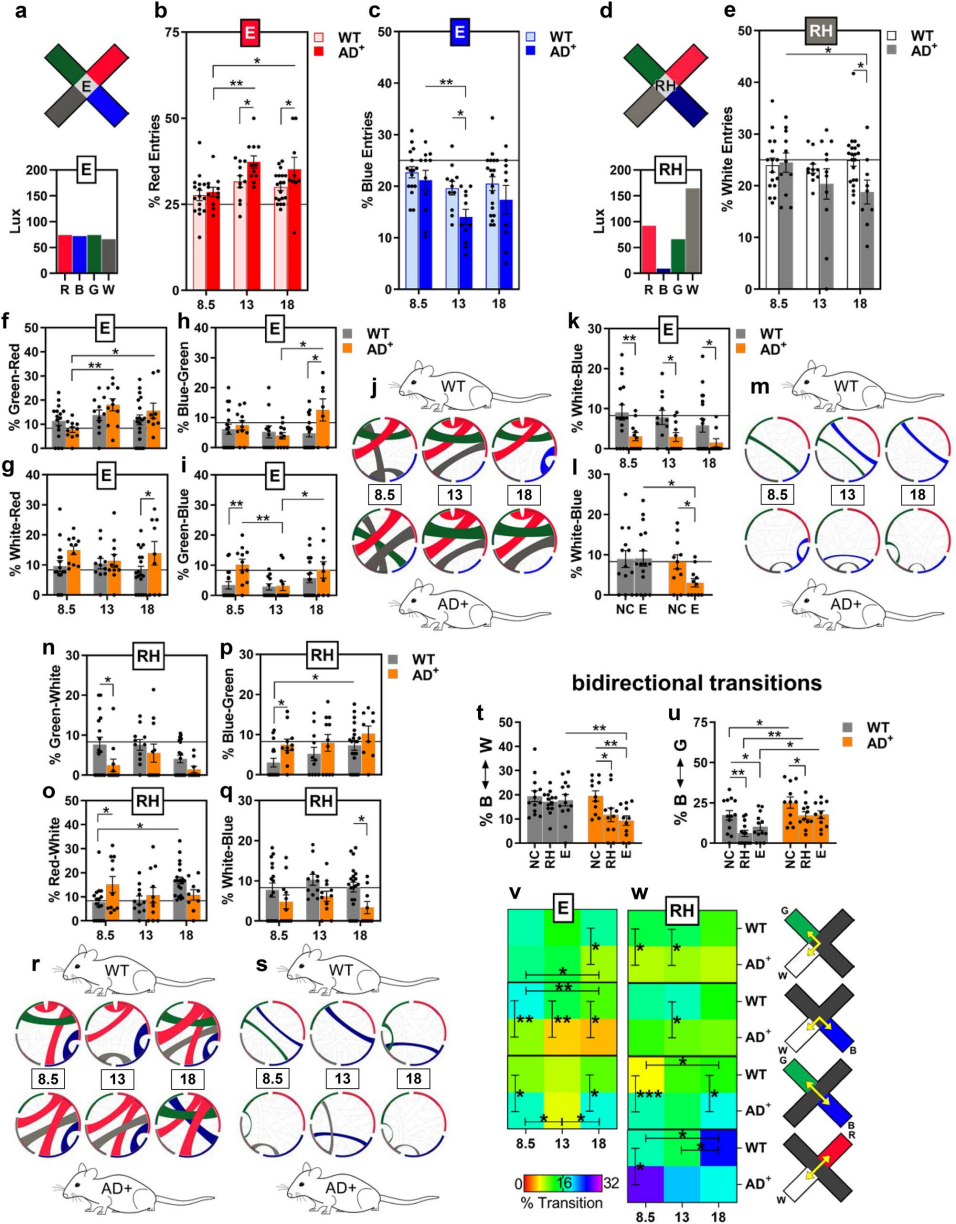
Entries and transitions of 8.5-, 13- and 18-month-old WT and AD^+^ mice in the ViS4M color mode. (**a**) Illuminance expressed in lux of the four colored arms for the E condition. (**b**,**c**) Percentage of entries in the red arm (**b**) and blue arm (**c**) of 8.5-, 13- and 18-month-old mice under the E condition. (**d**) Illuminance expressed in lux of the four colored arms for the RH condition. (**e**) Percentage of entries in the white arm of 8.5-, 13- and 18-month-old mice under the RH condition. (**f**–**m**) Percentage and frequency of unidirectional transitions between two colored arms under the E condition as follows: green to red (**f**), white to red (**g**), blue to green (**h**), green to blue (**i**), and white to blue (**k**). (**j**,**m**) Chord diagrams depicting the most frequent transitions (**j**) and the least frequent transitions (**m**) under the E condition. (**l**) Percentage of white-to-blue transitions of 8.5-month-old WT and AD^+^ mice under the No-Color (NC) and E conditions. (**n**–**s**) Percentage and frequency of unidirectional transitions between two colored arms under the RH condition as follows: green to white (**n**), red to white (**o**), blue to green (**p**), and white to blue (**q**). (**r**,**s**) Chord diagrams depicting the most frequent transitions (**r**) and the least frequent transitions (**s**) under the RH condition. (**t**,**u**) Percentage of bidirectional transitions of 8.5-month-old WT and AD^+^ mice under NC, RH and E conditions as follows: between blue and white (**t**), and blue and green (**u**). (**v**,**w**) Rainbow heat map illustrating the percentage of bidirectional transitions between green and white arms, blue and white arms, blue and green arms, and red and white arms (shown in right panel) under E (**v**) and RH conditions (**w**). Bottom left color gradient bar shows the range of percent transitions (from lowest in red to highest in purple). Mouse cohorts: 8.5-month-old WT (n = 16) and AD^+^ (n = 11); 13-month-old WT (n = 11–15) and AD^+^ (n = 11–15); 18-month-old WT (n = 19) and AD^+^ (n = 9). Group means, SEMs and individual data points are shown. * *p* < 0.05, ** *p* < 0.01, *** *p* < 0.001, **** *p* < 0.0001, by two-way ANOVA followed by *posthoc* Fisher’s LSD test.

We next assessed behavior related to color and/or luminance preference using the ViS4M in the three WT and AD^+^ mouse cohorts. WT and AD^+^ mice displayed different behavior in the distribution of time spent and percentage of entries for the colored arms, especially in the E and RH conditions (Figs. 1n,o, 2a–e; see extended data in Figs. S3–S8). Arm preferences were validated by comparison with the NC condition (Fig. S3a–c). A strong preference for the red arm, perceived in mice as black or dark grey (similar to protanopia), was detected for both genotypes as shown by higher percentages of time spent and entries in the red arm (Fig. 1n; extended data in Fig. S3–S8, panels a-b). This preference was seen across almost all ages regardless of intensity of red light, even when the red light absolute brightness (irradiance) was above the blue and green light intensities (RH condition, Fig. S5a,b) or the green and white light intensities (E condition, Fig. 1n). This result corroborates the dimming of red/orange lights that characterizes protanopes.

Under the E condition, the disparity in four color preferences becomes larger with aging in WT mice, and even more so, during disease progression in AD^+^ mice (Fig. 1n and Fig. S4a,b). The pattern change is especially noticeable in AD^+^ mice by reduced preference for the white arm (Fig. 1o, *p* < 0.05) and increased preference for the red arm (Fig. 2b, *p* < 0.05–0.01). Despite two-fold difference in irradiance of the white arm between the E and RH conditions, there was a similar behavior of reduced preference to the white arm in AD^+^ mice with disease progression (Fig. 2d,e, *p* < 0.05). Under E condition, compared to the red arm, the blue and white arms were least preferred and were substantially lower in the 13- and 18-month-old AD^+^ mice (Figs. 1n,o, 2b,c and Fig. S4b). Indeed, even though the blue arm is significantly brighter than the white arm (15-fold higher irradiance), AD^+^ mice showed equally reduced % time and % entries for these color arms (Fig. 1n). The blue and white arms share a spectrally similar activation of the true S-cones (Fig. S1b)^80^. Further, in the E condition, while the white and green arms had similarly low irradiance values (16 and 17 µW/cm^2^, respectively), there were significant increased preferences to the green arm as compared to the white arm in the AD^+^ mice versus age- and gender-matched WT controls (Fig. 1n and Fig. S4a,b). The green and white lights are spectrally different, since the green does not activate the true S cones (Fig. S1b). Hence, our data suggest that there are wavelength-specific visual impairments in AD^+^ mice, independent of luminance, as observed in response to the blue and white color stimuli.

### The ViS4M transition patterns reveal altered color discrimination in AD-model mice

We next analyzed % of the twelve unidirectional and % of the six bidirectional transitions between colored arms under the five illumination conditions (Fig. 2f–w; extended data for all conditions in Fig. S3d–o and Fig. S4–S8, panels c–f). These analyses provide unique information on transition patterns in mice due to color and luminance distinction. Under the E condition, there was an increase in % transitions from green-to-red (G–R) arms in AD^+^ mice during disease progression, which was apparent for the 8.5- as compared to 13- and 18-month old mice (Fig. 2f: Age: F_(2,71)_ = 4.275, *p* = 0.0177). No significant differences were observed relative to WT mice. In addition, % transitions from white-to-red (W–R) and blue-to-green (B–G) increased at a late disease stage in old AD^+^ mice vs. age-matched WT mice (Fig. 2g: Genotype: F_(1,71)_ = 5.524, *p* = 0.0215; Fig. 2h: Genotype x Age: F_(2,71)_ = 3.112, *p* = 0.0508). In terms of the green-to-blue (G–B) transition, there was a significant increase in the 8.5-month-old AD^+^ mice as compared to WT mice (Fig. 2i: Genotype: F_(1,71)_ = 5.789, *p* = 0.0187). At this age, using the NC condition paradigm, there were reduced % G-B transitions between the colored arms as compared to NC arms for both genotypes (Fig. S3i); this may indicate a specific effect of color on G-B transitions. Chord diagrams highlight the most frequent transitions for WT and AD^+^ mice, recapitulating the above-mentioned findings among other changes (Fig. 2j). The most striking difference between AD-model and WT mice was the substantial reduction in % white-to-blue (W–B) transitions observed in AD^+^ mice, which was revealed as early as 8.5 months of age (Fig. 2k: Genotype: F_(1,71)_ = 13.37, *p* = 0.0005). The reduction in % W-B transitions persisted during disease progression when compared with WT mice. NC condition paradigm, compared to the E condition, demonstrated color-related effects for the W-B transitions in AD^+^ mice, while no such effect is seen for WT mice (Fig. 2l: AD^+^, NC vs E, *p* = 0.048). Differences in W–B and G–B transitions between WT and AD^+^ mice are also visualized in chord diagrams of the least frequent transitions (Fig. 2m).

Under the RH condition, the main differences between WT and AD^+^ mice were seen early at 8.5 months of age. The % green-to-white (G–W; Fig. 2n: Genotype: F_(1,73)_ = 6.235, *p* = 0.0148) and red-to-white (R–W; Fig. 2o: Genotype x Age: F_(2,73)_ = 4.547, *p* = 0.0138) transitions were decreased and increased, respectively, in AD^+^ mice relative to WT mice. In addition, both % G-B (Fig. S5c: Genotype: F_(1,73)_ = 7.638, *p* = 0.0072) and % B-G (Fig. 2p: Genotype: F_(1,73)_ = 7.093, *p* = 0.0095) transitions were increased in 8.5-month-old AD^+^ mice when compared to WT mice, but were lower when compared to the NC condition for both genotypes (Fig. S3i,j). Under the RH condition, % W–B transitions were also impacted in AD^+^ mice but to a lesser extent than under the E condition (Fig. 2k), with reduced transitions that only reached significance at the age of 18 months (Fig. 2q for RH condition: Genotype: F_(1,73)_ = 9.834, *p* = 0.0025). The differences in unidirectional transitions under the RH condition between AD^+^ and WT mice over time are illustrated in chord diagrams for the most frequent (Fig. 2r) and least frequent transitions (Fig. 2s).

To account for a possible preference to transition in one direction rather than the other, we also compared bidirectional transitions between AD^+^ and WT mice. In this analysis, the visual impairment in AD^+^ mice was even more apparent (Fig. 2t–w). As previously suggested, the major change observed in AD^+^ mice was the substantial decrease of % combined W-B and B-W (B ↔ W) transitions observed under RH and E conditions, when compared to the NC condition (Fig. 2t: Condition, F_(2,44)_ = 4.415, *p* = 0.0179). Relative to WT mice, the reduction of % bidirectional B ↔ W in AD^+^ mice was detected as early as 8.5 months of age and persisted up to 18 months under the E condition (Fig. 2v: Genotype, F_(1,71)_ = 26.62, *p* < 0.0001). Under the RH condition, the % B ↔ W reduction was found later at 13 months of age as compared to WT mice (Fig. 2w: Genotype, F_(1,73)_ = 7.812, *p* = 0.0066). When compared to the NC condition, under color stimulation condition (RH and E conditions), both genotypes had reduced % bidirectional B ↔ G transitions (Fig. 2u: Condition, F_(2,44)_ = 7.787, *p* = 0.0013). Interestingly, the % B ↔ G transitions were much higher in AD^+^ mice than in WT under RH and E conditions (Fig. 2u: Genotype: F_(1,22)_ = 16.21, *p* = 0.0006). The increased % B ↔ G transitions were detected as early as in 8.5-month-old AD^+^ mice for all the conditions (Fig. 2v,w; extended data in Fig. S6–S8, f panel). Similarly, % G ↔ W and % R ↔ W transitions were also affected in AD^+^ mice, including as early as 8.5 months of age, with increased % transitions in all color modes versus NC condition (Fig. 2v,w; extended data in Fig. S3l,m, Fig. S6f, and Fig. S8f).

Collectively, the changes in % transitions described above for AD^+^ mice are likely to reflect perturbances in color distinction. Our next question was to determine if color vision as assessed by bidirectional transitions in the ViS4M could predict visual-cognitive performance as determined by % alternations.

### Color transition patterns predict alternation behavior of WT and AD^+^ mice in the ViS4M

**Figure 3:**
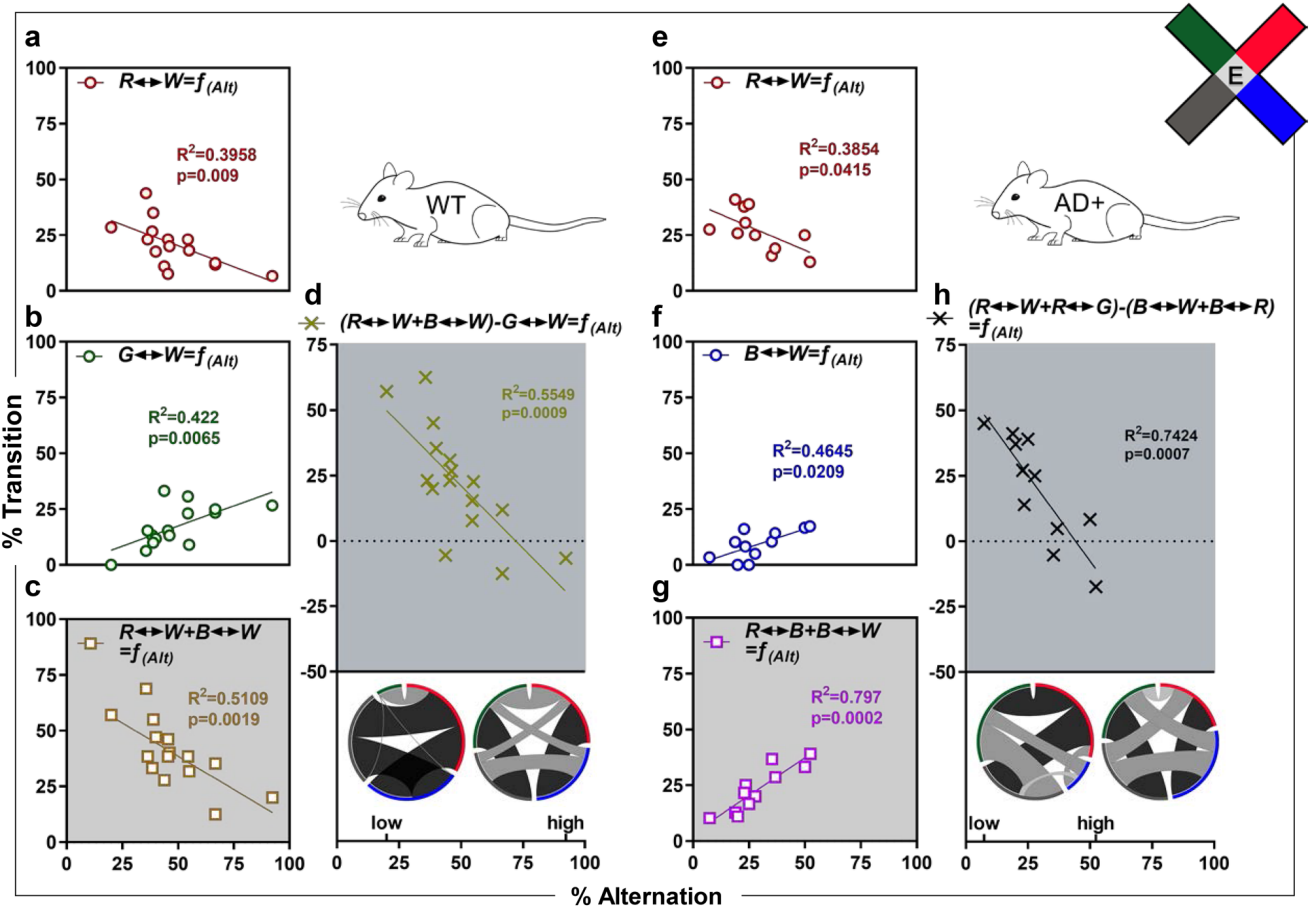
Correlation between alternation and color transitions in 8.5-month-old WT and AD^+^ mice under E condition. (**a**–**h**) Most significant linear regressions under E condition in WT (**a**–**d**) and AD^+^ mice (**e**–**h**) of the percentage of alternation with the percentage of single bidirectional transitions (**a**,**b**,**e**,**f**); or the percentage of positively paired bidirectional transitions (**c**,**g**); or the percentage of combined positively and negatively paired bidirectional transitions (**d**,**h**). (**d**,**h**) Inserts are chord diagrams depicting bidirectional transitions of the animals with the lowest (low) and highest (high) percentage of alternation. Mouse cohorts: 8.5-month-old WT (n = 16) and AD^+^ (n = 11). In all graphs, individual data points, fitted line, R-squared (R^2^) and *p* values are shown. The x-axis corresponds to the dependent variable (percentage of alternation) and the y-axis corresponds to the independent variables (predictors, percentage of transition).

Next, we performed linear regression analysis to assess correlations between the % bidirectional transition patterns and % spontaneous alternations in 8.5-month-old WT and AD^+^ mice. We show results obtained under E (Fig. 3 and Fig. S9a–j) and RH conditions (Fig. S9k–r). Under the E condition, the % alternations ranged from 20 to 96.7% in WT mice (Fig. 3a–d) and from 7.3 to 50.0% in AD^+^ mice (Fig. 3e–h). In both WT and AD^+^ mice, the % R ↔ W transitions negatively predicted visuocognitive-driven alternations in the ViS4M (WT: Fig. 3a, R^2^ = 0.3958, *p* = 0.009; AD^+^: Fig. 3e, R^2^ = 0.3854, *p* = 0.0415). These results demonstrate that deficient % alternation is observed in mice with the highest frequency of bidirectional transitions between white and red arms, regardless of the genotype.

In contrast, the % alternation improved in WT mice with increased % G ↔ W transitions (Fig. 3b; R^2^ = 0.422, *p* = 0.0065). In AD^+^ mice, the % alternation improved with increased % B ↔ W (Fig. 3f; R^2^ = 0.4645, *p* = 0.0209) or % R ↔ B transitions (Fig. S9f: R^2^ = 0.4484, *p* = 0.0242), or both combined (Fig. 3g; R^2^ = 0.797, *p* = 0.0002). Interestingly, although % B ↔ W did not individually correlate with % alternation in WT mice (Fig. S9d: R^2^ = 0.1954, *p* = 0.0865) when combined with % R ↔ W transitions there was inverse correlation with % alternation (Fig. 3c: R^2^ = 0.5109, *p* = 0.0019). In AD^+^ mice, an inverse correlation between % alternation was also found with % R ↔ W and R ↔ G transitions combined (Fig. S9j: R^2^ = 0.6203, *p* = 0.004). In addition, WT mice performed better in the ViS4M when G ↔ W transitions were more frequent than R ↔ W transitions (Fig. 3a,b and Fig. S9e) and reached their best performance when doing more G ↔ W transitions than the sum of R ↔ W and B ↔ W transitions (Fig. 3b–d: R^2^ = 0.5549, *p* = 0.0009). In both WT and AD^+^ mice, % alternation was unrelated to individual % B ↔ G transition (Fig. S9b, WT: R^2^ = 0.00022, *p* = 0.9563; Fig. S9g, AD^+^: R^2^ = 0.0225, *p* = 0.6598) or R ↔ G transition (Fig. S9c, WT: R^2^ = 0.04959, *p* = 0.4071; Fig. S9h, AD^+^: R^2^ = 0.2289, *p* = 0.1366).

Further, under the RH condition, we detected significant correlations between bidirectional % G ↔ W transition and % alternation in both WT and AD^+^ mice (Fig. S9l, WT: R^2^ = 0.5639, *p* = 0.0008; Fig. S9o, AD^+^: R^2^ = 0.5009, *p* = 0.0148). In this condition, the main factors that predicted alternation performance were % R ↔ W and % G ↔ W transitions individually (Figs. S9k,l and S9o), and % B ↔ W transitions combined with either % G ↔ W or % R ↔ W (Figs. S9m,n and S9q,r). Chord diagrams of bidirectional transitions for the worst and best performers in WT (E: Fig. 3d and RH: Fig. S9n) and AD^+^ mice (E: Fig. 3h and RH: Fig. S9r) reflected the influences of the different types of transitions on the alternation performances.

### Contrast sensitivity deficiency detected early in AD^+^ mice in the ViS4M

**Figure 4:**
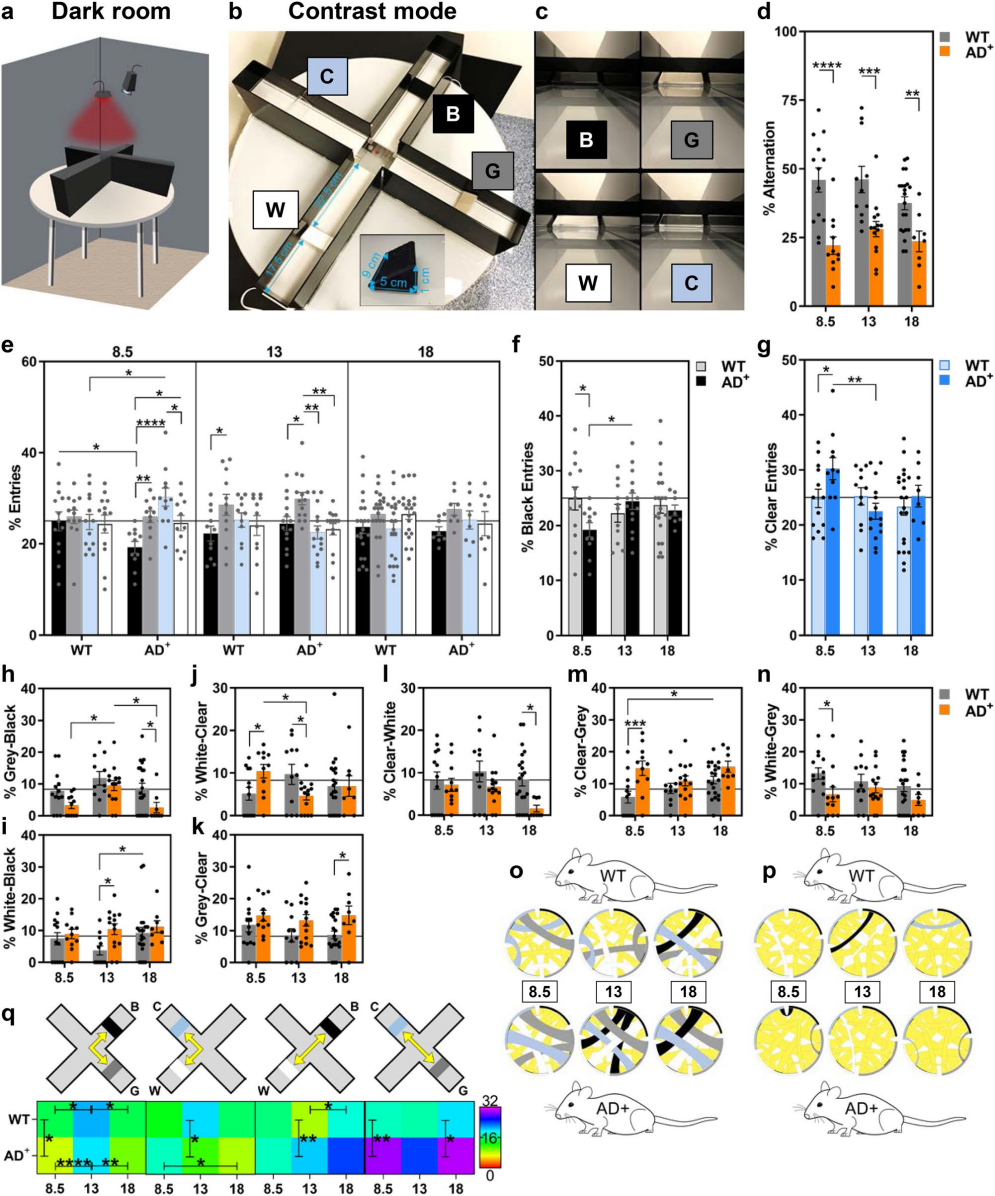
Alternation, entries and transitions of 8.5-, 13- and 18-month-old WT and AD^+^ mice in the ViS4M contrast mode. (**a**) Apparatus set-up in a dark room under red light illumination with a video camera. (**b**,**c**) Images of the ViS4M with the four objects: B = black, C = clear, G = Grey and W = white. (**b**) Dimensions of the objects and positioning within the arms of the ViS4M. (**c**) Views of the objects when entering the arms of the maze. (**d**) Percentage of alternation. (**e**) Percentage of entries in the arms containing different objects. (**f**–**g**) Percentage of entries in the arm containing the black object (**f**) and the clear object (**g**). (**h**–**p**) Percentage and frequency of unidirectional transitions between two arms as follows: grey to black (**h**), white to black (**i**), white to clear (**j**), grey to clear (**k**), clear to white (**l**), clear to grey (**m**), and white to grey (**n**). (**o**–**p**) Chord diagrams depicting the most frequent transitions (**o**) and the least frequent transitions (**p**). (**q**) Rainbow heat map illustrating the percentage of bidirectional transitions between arms with grey and black objects, white and clear objects, white and black objects, and grey and clear objects (shown in top panel). The color gradient bar shows the range of percent transitions (from lowest in red to highest in purple). Mouse cohorts: 8.5-month-old WT (n = 13; 9 males and 4 females) and AD^+^ (n = 11; 8 males, 3 females); 13-month-old WT (n = 11; 6 males, 5 females) and AD^+^ (n = 14; 7 males, 7 females); 18-month-old WT (n = 19; 11 males, 8 females) and AD^+^ (n = 9; 5 males, 4 females). Group means, SEMs and individual data points are shown. * *p* < 0.05, ** *p* < 0.01, *** *p* < 0.001, **** *p* < 0.0001, by two-way ANOVA followed by *posthoc* Fisher’s LSD test.

We next explored contrast sensitivity in mice using the ViS4M. In contrast mode, testing was conducted under red light illumination (dark) and no colored lights were used within the apparatus (Fig. 4a). The ViS4M was configured by introducing an object positioned halfway between both ends of each arm. The four objects all had the same shape but four different grayscale shades: white, black, grey and clear (Fig. 4b). In combination with the white floors and black walls, the ability to visually detect the four objects was likely dependent on contrast sensitivity (Fig. 4c). The measured luminance ratio of the black object against the black walls is 1.06 (minimal to no contrast), the gray object against the black walls is 6.00, the white object against the black walls is 9.69 (high contrast against the black walls but minimal contrast with the white floor), and the clear object against the black walls is 6.56.

Similar to the color mode, in the contrast mode, WT mice showed a propensity to spontaneously alternate between the arms with the different objects (Fig. 4d). The alternation behavior was dramatically reduced, from 46 to 22%, in AD^+^ versus WT mice as early as 8.5 months of age (Fig. 4d: Genotype, F_(1,72)_ = 39.88, *p* < 0.0001). The magnitude of reduction between WT and AD^+^ mice was even higher in 8.5- (52%) than 18-month-old (37%) mice, probably due to decreased alternation with age in WT mice (*p* = 0.059; Fig. 4d: Genotype, F_(1,72)_ = 39.88, *p* < 0.0001). In WT mice, there was no major preference for a particular arm, as shown by the % entries in each arm, besides a slight preference for the grey arm observed at 13 months of age only (Fig. 4e). Arm preferences were more obvious in AD^+^ mice, with lower % entries to the black arm and higher % entries to the clear arm as compared to WT mice observed at 8.5 months of age (Fig. 4e–g; *p* < 0.05).

Next, differences in arm transitions were identified between WT and AD^+^ mice, further supporting impaired contrast sensitivity in diseased mice (Fig. 4h–q). When compared to WT mice, AD^+^ mice showed decreased % grey-to-black transitions that reached significance at 18 months of age (Fig. 4h: Genotype, F_(1,72)_ = 8.161, *p* = 0.0056) and significantly increased % white-to-black transitions at 13 months of age (Fig. 4i: Genotype, F_(1,72)_ = 4.868, *p* = 0.0306). In addition, AD^+^ compared to WT mice showed differences in % white-to-clear transitions, at both 8.5- and 13-months of age (Fig. 4j: Genotype x Age: F_(2,72)_ = 4.397, *p* = 0.0158). At a later stage, AD^+^ mice exhibited increased % grey-to-clear and decreased % clear-to-white transitions (Fig. 4k,l; *p* < 0.05). Interestingly, the 8.5-month-old AD^+^ mice showed a significant increase in % clear-to-grey transitions (Fig. 4m: *p* = 0.0004) along with lower % white-to-grey transitions (Fig. 4n: *p* < 0.05). Chord diagrams of the most frequent (Fig. 4o) and least frequent unidirectional transitions (Fig. 4p) reflect the differences between WT and AD^+^ mice. Finally, analysis of bidirectional transitions revealed differences in contrast sensitivity between WT and AD^+^ mice that pertained to the discrimination of grey and black (Genotype: F_(1,72)_ = 7.818, *p* = 0.0066), white and clear (Genotype x Age: F_(2,72)_ = 4.084, *p* = 0.0209), black and white (Genotype: F_(1,72)_ = 6.098, *p* = 0.0159), and grey and clear (Genotype: F_(1,72)_ = 15.89, *p* = 0.0002) shades (Fig. 4q). Significant changes with age in bidirectional transitions were observed with similar patterns in both WT and AD^+^ mice between grey and black arms, between white and clear arms in AD^+^ mice only, or between black and white arms in WT mice only (Fig. 4q).

## Discussion

In the present study, we developed a behavioral visual-stimuli four-arm maze (ViS4M) to assess color vision and contrast sensitivity in mice and applied it in the context of normal aging and AD. This versatile maze allows visual-behavioral assessments across an array of mesopic and photopic illuminations, with predefined wavelengths and accessories for color and contrast conditions. We analyzed a plethora of parameters including alternation patterns, number of arm entries, time spent in each arm, as well as unidirectional and bidirectional transition profiles. We found that (1) the ViS4M is a sensitive tool to assess specific aspects of vision such as color preferences and discrimination and contrast sensitivity in mice; (2) the newly developed device reveals early AD-related impairments in color vision and contrast sensitivity; (3) color discrimination guides spontaneous behavior in mice; and (4) AD-specific cognitive impairments could be attributed to deficits in color vision and contrast sensitivity.

We report the phenotypic assessment of color vision and contrast sensitivity in WT and AD-model mice using the ViS4M. In other behavioral tests such as the Y-maze, spontaneous alternation measures the working memory^104^. Our data suggest that the colored arms or contrasting objects provide cues that mice use to sequentially explore the maze. This trait in mice implies a certain ability to discriminate between colors and grayscale shades, in addition to their cognitive capacity (working memory). AD^+^ mice were largely incompetent to successfully alternate at all ages and in all conditions, which could be attributed to impairments in color and illumination distinction or contrast sensitivity, alone or combined with cognitive deficits. Thus, the ViS4M should provide a sensitive screening tool to jointly assess color, luminance, contrast vision and working memory associated with color or contrast stimuli.

Whether structural and functional pathology of AD patients and AD-model mice appears first in the retina is an open debate^9,12,29–31,33,36–38,53,105–109^. In humans, a wide range of visual disturbances have been reported in MCI and AD patients, including problems with color vision and contrast sensitivity, which could be attributed to AD pathology in the retina^11,33,45–61^. We and other groups have previously shown that the same molecular hallmarks of the AD brain occur in the retina of AD animal models^6,33^ and that they can be detected in parallel or prior to their detection in the brain^9,14,15,38,41^. Here, we report alternation deficits as early as 8.5 months in AD^+^ mice, months before cognitive deficits could be detected. Indeed, in our previous studies, we did not find significant cognitive impairment in these transgenic murine models using Y-maze, Morris water maze, or Barnes maze prior to 10 months of age^100,102,103^. Others have shown similar data in the Morris water maze^110^, T-maze^111^, and radial arm water maze^112^. To our knowledge, only a few studies have reported mixed results regarding earlier cognitive deficits in double transgenic APP_SWE_/PS1_∆E9_ mice. Cao et al. have shown a slight impairment in the acquisition phase (learning) of the Morris water maze, but the % alternation in the T-maze (working memory) was normal at 6 months^113^. In the contextual fear conditioning test, long-term memory (24 h) was largely reduced in 6-month-old AD^+^ mice, while short-term memory (1 h) was unaffected^114^. Overall, we can say with confidence that hippocampal-based spatial working memory is intact or minimally impacted in young adult (8.5 months) APP_SWE_/PS1_∆E9_ mice used in this study.

Since we cannot precisely measure the contributions of vision deficits and memory impairment in the worsened ability to alternate between the four colored arms in AD^+^ mice, it is possible that the ViS4M is more sensitive and efficient in detecting memory deficits. However, in the absence of color or contrasting objects, there were no significant differences in alternation, pointing to color and contrast vision defects in younger AD^+^ mice. In addition, in 8.5-month-old AD^+^ mice, the % alternation could almost be entirely predicted (R^2^ = 0.797) by the increase of the combined B ↔ W and R ↔ B transitions, suggesting minimal effect of cognitive impairment and largely a visual impairment. Further, we did not find such a strong correlation between alternation and arm transitions in older AD^+^ mice when cognitive deficits were detected with other behavioral paradigms (data not shown). To summarize, our results suggest that the lower alternation in 8.5-month-old AD^+^ mice could depend mostly on AD pathology in the retina and reflect vision impairment. As the disease progresses, the low alternation seen in older mice could reflect AD pathology in the retina and hippocampus resulting in a combination of both visual and cognitive impairments. We cannot rule out that earlier vision deficits may occur in these AD^+^ mice since younger mouse cohorts were not tested; hence, future studies ought to explore how early these visual deficits can be detected.

It is likely that mice use visual cues to discriminate between the arms of the ViS4M to explore the maze in sequence in at least two of our conditions (RH and E). According to the nature of our light stimuli, arm distinction may be driven by color or luminance differences. There are two aspects of the mouse color visual system that we controlled in ViS4M: the spectral activation of mouse opsins and the retinotopic distribution of mouse cones. Mice are dichromats and have two cone types in their retina: S-cones with a peak sensitivity at 360 nm tuned to sense UV light^78,115^, and M-cones with peak sensitivity at 508 nm, sensitive to green light. Importantly, it has been shown that the S-opsin has an absorbance sensitivity that extends to 450 nm^116,117^. Color discrimination in the ViS4M requires that at least some of the color lights stimulate the true S-cones and M-cones differently. Studies using blue, green and red lights, regardless of the response under investigation, often discuss their results in terms of M-cones, rods and melanopsin, citing lack of sensitivity of S-cones^118^. However, a robust electrophysiological study has shown that blue light (460 nm) is able to stimulate true S-cones and elicit a sustained response that is distinct from that of M-cones, rods and melanopsin^80^. Indeed, the intensities of our blue and white stimuli are estimated to be in the effective range of true S-cone activation, allowing for color discrimination.

The other aspect of the mouse color visual system is the asymmetric distribution of cones across the retinal topography. M-cones co-express S-opsin and form an opposing gradient of expression along the dorsoventral axis. True S-cones, expressing only S-opsin, are sparse in the dorsal retina but make up to 30% of the cone population in the ventral retina^87^. A collection of recent electrophysiological, behavioral and imaging studies point to the ventral retina as the source of color vision, thus limited to the upper visual field^88,119^. Nevertheless, head-fixed mice stimulated in the upper visual field were shown to be equally or even more sensitive to chromaticity contrast closer to the dorsoventral transition zone (+ 10° vs. + 30°)^88^. Taking this into consideration, the ViS4M maze apparatus was designed such that lights from the floor homogenously diffused into the entire arm and reflected from the clear plastic ceilings. In addition, since the mice are freely exploring the maze, they often move their head in all directions and their eye topography changes constantly. Therefore, we assume that the ViS4M lights provided full-field illumination of the retina and stimuli for color vision.

Mice are more sensitive to luminance than chromaticity variations^88^. Since mice lack L-cones, in the ViS4M the red arm should be perceived as an achromatic dark and likely safe area. Not surprisingly, both WT and AD-model mice had an overall preference for the red arm. However, while mice spent more time in the red arm with concomitantly increasing intensity of the four stimuli (L vs M vs H conditions), they spent the most time in the red arm when only the intensity of the red stimulus was increased compared to the other three arms (RH condition). Moreover, M-cones were likely stimulated by the red stimulus, though to a lower extent than by the blue, green and white lights, but still adding a chromatic component to the discrimination of red light. We speculate that mice switched from a spatial strategy, possibly using whiskers (L condition), to a visual strategy (M, H, RH and E conditions) with higher red-light intensities. It has been shown that mice respond to bright red light^120^. For instance, at a higher threshold than blue and green lights, red light is able to elicit non-image forming responses such as photoentrainment and pupillary light reflex^118,121^. Under E condition, we tested the effect of high blue irradiance compared to similarly low irradiance in the three other color arms. Here again, the expected response was the avoidance of the bright light stimulus as shown by a dramatic decrease in time spent in the blue arm. Aversion to blue light, as opposed to yellow light, has been reported in WT mice and seems to involve the pupillary light reflex and likely cones and ipRGCs^122,123^. The previous data show not only that the ViS4M is able to detect these simple innate responses to dark (red) and bright (blue under E condition) stimuli, but also that these responses are likely not affected in AD^+^ mice.

However, under the E condition, AD^+^ mice exhibited avoidance of the dim white arm to the same extent as the bright blue arm, which worsened during disease progression in the older ages starting at 13 months of age. Interestingly, a similar response to the white light was observed under RH condition, where white and blue lights have similar intensities. Under RH condition, 18-month-old AD^+^ mice visited the blue arm more often and for longer time than the white arm. WT mice did not show preferences for either the white, blue or green arm. Moreover, the observation that the avoidance of the white arm in AD^+^ mice is closely related to the low bidirectional B ↔ W transitions suggests a deficiency to discriminate between these two arms. Altered brightness perception alone cannot explain the inability to discriminate between the blue and white arms since it occurred with either dim or bright blue stimulus. This is further supported by the opposite effect of B ↔ W transitions on alternation performance in both WT and AD^+^ mice. Indeed, in 8.5-month-old WT mice, increased alternation correlated with increased or decreased B ↔ W transitions under RH and E conditions, respectively, while AD^+^ mice showed the opposite. Altogether, our results suggest a strong role for color vision with respect to discrimination between the blue and white stimuli.

The arm transitions profiling of AD^+^ mice revealed that under E condition, the B ↔ W transitions of AD-model mice were substantially decreased in 8.5-month-old mice when compared to NC condition and at all ages when compared to age-matched WT mice. Moreover, increased performance (alternation) of 8.5-month-old AD^+^ mice was positively correlated to the amount of B ↔ W transitions. These data support reports showing that patients with AD have a selective deficit in blue hue discrimination assessed with clinical color vision tests^66^. Most importantly, blue hue discrimination deficiency has been found more frequently in the early stages of AD^49,59,65,67^ or could be independent of AD severity^66^, which corroborates our finding of early and stable lower B ↔ W transitions in all three age groups of AD^+^ mice. Blue color discrimination impairment was also reported in patients with neurodegenerative disorders such as multiple sclerosis, Parkinson’s disease, Huntington’s disease and Gilles de la Tourette syndrome^124–129^. Though not specific to AD, our data support the relevance of monitoring patients with acquired blue color blindness; however, to our knowledge there are no studies of blue color blindness acquired early in life and AD diagnosis.

We cannot preclude the primary visual cortex from being involved in the visual deficiencies in AD, since AD pathologies are detected in these regions as well^68,130,131^. However, the presence of retinal Aβ plaques together with degeneration of RGCs^17,31,132,133^, including melanopsin-containing intrinsically photosensitive RGCs (ipRGCs)^11^, and optic nerve atrophy^134^ make the retina a prime candidate in the blue hue distinction defect of AD patients. Moreover, optic nerve damage caused by acute or multiple sclerosis-associated optic neuritis have been shown to potentially cause blue color blindness as well^135^. In our study, the B ↔ W transitions also decreased significantly during normal aging in 18-month-old when compared to 8.5-month-old WT mice but remained higher than in age-matched AD^+^ mice. More importantly, this decrease in B ↔ W transitions was not associated with a decline in arm alternation, showing that the ViS4M is sensitive to detect even slight color distinction impairments. In humans, blue-yellow defects have been shown to be common among the aged population and to become more prevalent with increasing age^136^. Interestingly, the authors note that most subtle aging-related color vision abnormalities are likely to go unnoticed, further supporting our findings.

Under E and RH conditions, the B ↔ G transitions were much lower in both WT and AD young mice when compared to NC condition, suggesting a lower ability to discern between these two arms, independent of the brightness of the blue stimuli. In addition, under E condition, a marked decrease in G ↔ W transitions was observed in older AD-model as compared to WT mice, while under RH condition, it was seen starting as early as 8.5 months of age. Interestingly, as opposed to blue and white stimuli, the ability to discriminate between white and green lights highly predicted the likelihood to alternate in both WT and AD^+^ mice under RH condition. It should be noted that under RH condition, the performance of WT mice was tightly correlated to the ability to transition between green and white arms as much as between blue and white arms, suggesting that the white can stimulate a strong true S-cone response. Notably, under the E condition where green and white arms have equal irradiance, AD^+^ mice exhibited very different color preference behavior between white and green. This distinct behavior for white versus green in AD-model mice further supports color vision deficits. Overall, the AD-model mice seem to have wavelength-specific visual impairments as further shown by the lower preference for the white and blue arms, which are likely dependent on S-opsin stimulation.

Even though the B ↔ G transitions decreased in AD^+^ mice when compared to NC condition, suggesting a lower aptitude at distinguishing between blue and green arms, they were still much higher in AD^+^ than in WT mice under RH and E conditions. We may also attribute this effect to the hyperactivity of this mouse strain model of AD^137^, as shown by the average speed, longer distance traveled, and a higher number of total entries in the ViS4M, especially in young mice. Indeed, in our setup the blue and green arms were facing each other and AD^+^ mice were more likely to run straight and consequently make a higher number of these transitions. It should be noted that this effect is also likely due to low B ↔ G transitions in WT mice. Transitions between the two other facing arms, white and red, were among the most frequent transitions in both WT and AD^+^ mice and were significantly higher in AD^+^ mice under RH condition. We cannot rule out that the higher speed of AD^+^ mice in the ViS4M affected their ability to alternate. Indeed, many successive B ↔ G and R ↔ W transitions would easily decrease the % alternation. However, it is unlikely that such behavior would specifically affect only one type of all the other transitions, and we can safely state that the decrease of B ↔ W transitions under E condition is the consequence of a visual deficit rather than speed of AD^+^ mice. Altogether, these observations support our idea that early visual impairments manifest in AD^+^ mice prior to cognitive decline, since the higher speed, distance traveled, and total entries occurred especially in 8.5-month-old mice. Nevertheless, this type of behavior could be a confounding factor in the detection of color distinction (arm transitions). We overcame this caveat by adding the NC condition by switching the position of the colored LED lights illuminating the arms of the ViS4M. Future studies should address this question under alternate illumination conditions.

The underlying cause of the color deficit in AD^+^ mice remains obscure and was beyond the scope of this study. Indeed, the chosen light stimuli in the ViS4M do not allow us to separate the contribution of S-opsins versus M-opsins. Nevertheless, the blue and white stimuli are likely to activate both M and true S-cones, at different levels, giving rise to their discrimination based on chromaticity, depending on their retinotopic location. Uncommon color opponency pathways, in combination with the asymmetrical distribution of S- and M-opsins, further complicate color vision in mice. And in addition to traditional cone opponency, we cannot rule out a role for cone-rod opponency^76^, which occurs in the mesopic range, as used in this study. Other color vision schemes include unselective-cone pathways and alpha-like RGCs near the transition zone^138^, or the M5-type ipRGC^139^. Moreover, S-cones, indispensable for color vision, may also play a role in the coding of dark contrast of achromatic stimuli^83^ and in non-image forming response such as the pupillary light reflex^80^. Even though brightness was a confounding factor, our findings support that color (wavelength) discrimination had a key role in mouse visual behavior and in AD^+^ mice deficiency. Future studies may take advantage of the modulatory features of ViS4M and replace blue with UV LED to confirm behavior directly related to S-cone activation and color discrimination.

Along with the decrease in color perception, loss of contrast sensitivity is among the earliest symptoms documented in AD patients^33,140^. In the present study, we show that in contrast mode, AD^+^ mice are incapable of alternating between the four arms of the ViS4M starting as early as 8.5 months, when working memory is not yet impacted by AD pathology (see discussion above). This result suggests that AD^+^ mice cannot discern some of the objects against the walls and floor background and supports an early deficit in contrast sensitivity. Importantly, AD^+^ mice exhibited a significant decrease in % entries to black-object arm at 8.5 months of age as compared to the WT mice. The nature of the defect is currently unclear but could be related to the minimal contrast of the black object against the black arm wall background. Further, the white object may be viewed by the mouse as a continuation of the white floor, while the gray and clear objects have contrast against both the white floor and the black walls. This result can explain the preference to these object arms, noted in the older 13- and 18-month-old mice, even when contrast sensitivity is reduced. However, these results provide evidence for application of the ViS4M to study contrast sensitivity, which will be the subject of future investigation.

In conclusion, we demonstrated the potential of the novel ViS4M to detect and characterize specific changes of color vision and contrast sensitivity that occur in AD^+^ mice. For instance, our findings revealed early deficits in white-blue and white-green distinction in AD^+^ mice and suggest a possible alteration of the perception of white light. These results are very exciting in view of a recent study attributing a new role for melanopsin in unique white perception^141^. The ViS4M is an amenable solution with minimal caveats that requires only 5 min of testing (per animal and per light intensity condition), as opposed to current methodologies necessitating long sessions of training and thousands of trials. Unlike previous behavioral tests, the novel ViS4M is sensitive and easy to set up, allowing mice to move freely without introducing stress. Importantly, the test relies entirely on innate exploratory behavior and does not require a pre-training phase nor rewards. Moreover, this test can simultaneously assess cognitive and visual functions.

This ViS4M offers flexibility and various possibilities to undercover behavioral deficits while controlling confounding factors related to the complex color vision system. Careful and strategic design of experiments in combination with the large catalog of transgenic mice related to color vision, and especially the trichromatic vision mouse^75^, will allow vision and behavioral scientists to make discoveries in the field of murine models that could potentially be translated to improved human visual testing and diagnosis. The American Academy of Ophthalmology recommends a baseline comprehensive eye exam for healthy adults with no symptoms or known risks at age 40 (https://www.aao.org/eye-health/tips-prevention/midlife-adults-screening). However, this vision screening does not include color or contrast vision. While noninvasive retinal amyloid imaging as a potential screening tool for AD seems appropriate, additional color and contrast vision tests^53^ would be highly relevant to detect early symptoms beyond the molecular risk factors that could be easily implemented to assess AD-associated functional defects.

## Methods

### Mice

The double transgenic B6.Cg-Tg(APP_SWE_/PSEN1_∆E9_)85Dbo/Mmjax hemizygous (AD^+^) mouse strain [RRID:MMRRC_034832-JAX] was used in this study. AD^+^ mice were initially purchased from the Mutant Mouse Resource and Research Center (MMRRC) at the Jackson Laboratory, then bred and maintained at Cedars-Sinai Medical Center. The mouse colony was housed in a humidity- and temperature-controlled (21–22 °C) vivarium on a 12:12-h light/dark cycle (lights on at 8:00am; lights off at 8:00 pm) with free access to food and water. Non-transgenic wild-type (WT) littermates served as controls. Three different cohorts of mice were tested, each representing a different age: 8.5-month-old WT (n = 16; 9 males and 7 females) and AD^+^ (n = 11; 8 males and 3 females), 13-month-old WT (n = 13; 6 males and 7 females) and AD^+^ (n = 15; 7 males and 8 females), and 18-month-old WT (n = 19; 11 males and 8 females) and AD^+^ (n = 9; 5 males and 4 females).

All experiments were conducted in accordance with Cedars-Sinai Medical Center Institutional Animal Care and Use Committee (IACUC) guidelines under an approved protocol.

### Visual-stimuli four-arm maze (ViS4M) apparatus and accessories

The backbone of the custom-made ViS4M apparatus consists of an × -shaped enclosure built with 15 cm-high black plexiglass walls attached to a glass base. Each arm is perpendicular to the two adjacent arms and is 45 cm long and 10 cm wide. Removable transparent floor plates can be inserted at two different levels (5.5 cm or 11 cm high from the glass base) in each arm and in the center of the ViS4M separately. Below each floor level, small track brackets made of plexiglass are attached to the walls of the ViS4M and allow white translucent acrylic plates to be inserted. The four arms possess individual plastic transparent covers with perforated holes to help with breathing.

#### Color mode testing

Four light-emitting diode (LED) sources provide monochromatic light at a single wavelength λ (Red: 628, FWHM 17 nm; Green: 517, FWHM 31 nm; Blue: 452, FWHM 22 nm; and White: λ_1_ 441, FWHM 19 nm and λ_2_ 533, FWHM 104 nm). The white LED is made of a blue-emitting diode that also excites a yellow-emitting phosphor—cerium doped yttrium aluminum garnet (Ce:YAG-Y_3_Al_5_O_12_) crystals—embedded in the epoxy dome. Spectra of the light sources were determined using an Ocean Optics USB2000 spectrometer and further validated with a Sekonic C700-U spectrometer. LED strip source consists of an array of surface-mounted device (SMD) 3528 LED chips evenly spaced in four rows (27 LED chips per row). Each chip has an emission angle of 120°. Each LED source is individually inserted in each arm of the ViS4M, directly on the glass base. Above them, the white translucent acrylic plates and floor plates are positioned on the top level. The arrangement of the LED chips in combination with the white translucent plates is optimized to allow the light to diffuse equally in all directions and create a spatially homogenous stimulus. No individual LED spots were discernable. The clear plastic covers were installed on top of the arms and further homogenized the light stimuli into the arms by reflection. Each LED source was connected to a LED single color dimmer wirelessly controlled with an individual remote device. The dimmer used pulse width modulation (PWM) technology to increase or decrease brightness of the light stimuli. Five sets of intensities were tested (low L, medium M, high H, red-high RH and equal E). The L, M and H intensities were selected to cover the range of illuminance that could be obtained with the red source (L: 6 lx, H: ~ 100 lx). The illuminances of the three other sources were determined by a similar setting than the red source using the remote controls. The RH condition was obtained by selecting the H intensity for the red source, while the three other sources were set on L intensity. For the E condition, we increased the illuminance of the blue source compared to RH to achieve equal luminance for the four sources, set to the transition mesopic/photopic range (~ 3 cd/m^2^).

Incident illuminance (lux) was uniform across each arm and measured for each color in a dark room using a Sekonic L-308S light meter according to the manufacturer’s instructions (lumidisc, EV mode and ISO 100). For each measurement, the flat surface of the light meter sensor was positioned facing and about 2 cm above the floor (mouse eye level) at a locus situated in the middle of the arm. The positioning of the sensor and locus of measurement were chosen for consistency and practicability, assuming the light coming from each LED source diffused in all directions equally into its respective arm. The recorded exposure values (EV) were converted to illuminance units (lux) and luminance units (cd/m^2^) using the relationships lux = 2.5 × 2^EV^ and cd/m^2^ = 2^(EV-3)^, respectively. Since mesopic and photopic visions occur in the luminance range of 0.001–3 cd/m^2^ and above 10 cd/m^2^, respectively, our light stimuli can be considered as mesopic or photopic, depending on the condition, but mostly in the overlapping transition state between the two. Irradiance (μW/cm^2^) and log photon flux (log_10_ photons/cm^2^/s) were calculated from illuminance (lux) using the Rodent Toolbox V1, freely available at https://www.ndcn.ox.ac.uk/team/stuart-peirson. In addition, for each condition, the effective quantal flux (in log_10_ effective photons/cm^2^/s) of each light stimulus was estimated for each opsin photopigment by weighting spectral irradiance according to pigment spectral efficiency. For these estimations, the Rodent Toolbox V1 considers the spectral irradiance, the pigment spectral sensitivity^142^ and the mouse lens transmission^143^. All measures and estimations are shown for each stimulus and each condition in Fig. S1c.

All testing took place in the dark in the Biobehavioral Core facility during the last third of the light cycle (between 4:00 pm and 8:00 pm). The sole light source was provided by the illuminated arms of the ViS4M. Mice were brought to the testing room and left undisturbed to habituate to the dark room for 30 min. At the end of the acclimation period, light intensities were selected for the four arms of the ViS4M. Mice were individually placed in the center of the maze through the opened top, left to freely explore the maze for 5 min, and then returned to their home cage. The five conditions of testing were carried out on five consecutive days in the following chronological order: L, M, H, RH and E. Due to high mortality rate, especially among old AD^+^ mice, and to minimize fatigue and stress, L and H conditions were not tested in the 18-month-old cohort of mice. The omitted conditions were selected based on minimal to no changes detected in the younger cohorts. Hence, 18-month-old mice were tested on three consecutive days in the following chronological order: M, RH and E conditions. In addition, the 8.5-month-old cohort of mice was tested for a sixth condition—no-color (NC) condition—that was carried out prior to the other five conditions. For this condition, the arm LED sources were not lit, and testing was carried out in a dimly lit room.

#### Contrast mode testing

Four objects positioned inside the arms of the ViS4M are used in the contrast mode. The objects have identical shape of a right-angled triangular prism but different shades (black, grey, clear, and white), creating different contrasts with the black walls and white floors of the maze. The objects are individually placed in the middle of each arm on their adjacent side (base, 5 cm) with their opposite side (height, 1 cm) facing and 22.5 cm away from the entry of the arm. The hypotenuse faces the end of the arm.

In our study, the floor plates were positioned on the bottom level with the semi-transparent white plates placed below them. None of the LED sources were used and testing was carried out under dim red-light illumination (mesopic, ~ 0.1 cd/m^2^) in the Biobehavioral Core facility during the last third of the light cycle (between 4:00 pm and 8:00 pm). Mice were brought to the testing room and left undisturbed to habituate for 30 min. At the end of the acclimation period, mice were individually placed in the center of the ViS4M, allowed to freely explore the maze for 5 min, and then returned to their home cage. Contrast mode testing was conducted once and last after color mode testing.

#### Testing analysis

All sessions were videotaped with a camera positioned above the ViS4M for further analysis. Total distance traveled (entire ViS4M) and time spent in each arm were measured from the video recordings using ANY-maze behavior tracking software 6.1 (www.stoeltingco.com/anymaze/video-tracking/software.html). Sequences of arm entries were manually recorded from the video recordings by an experimenter blind to the genotype of the mice. Total entries, percentage of entries in each arm, percentages of unidirectional and bidirectional transitions between arms, and percentage of alternation are all derived from the sequences of arm entries. The percentage of alternation is defined as the sequential visit of the four different arms without returning to a previously visited arm relative to the total number of sequences of four arms (total number of arm entries minus 2).

### Barnes maze test

The Barnes maze test was carried out on the 13-month-old cohort of WT and AD^+^ mice to assess hippocampus-based spatial learning and memory, as previously described^101^. Briefly, mice were first trained for 4 days to locate an escape box among 20 similar-looking holes, equally spaced along the perimeter of a flat circular platform. A bright light illuminated the center of the Barnes maze and served as a mild aversive stimulus to promote the search of the escape box. Training was followed by a 2-day break with no exposure to the maze. Mice were then tested for memory retention on day 7. Mice underwent 3 trials per day each day of training/testing. Mouse performances (latency to find the escape box and number of incorrect entries) were recorded for each trial and averaged. In addition, chord diagrams were generated to visualize the search strategy of the mice.

### Noninvasive in vivo retinal plaque imaging

Live retinal Aβ plaque imaging in AD^+^ mice was conducted at the age of 8.5 and 13 months, according to our previously described protocol^9,10,34^. Briefly, eyes of anesthetized AD^+^ mice (70 mg/kg Ketamine and 0.5 mg/kg Dexmedetomidine) were dilated and imaged following systemic administration of the fluorescent compound curcumin (Sigma-Aldrich; 7.5 mg/Kg; IV injection once a day for 3 consecutive days). Movie clips of the retina were repeatedly captured using Micron III retinal imaging microscope for rodents (Phoenix Research Laboratories, San Ramon, CA), with identical light exposure time and intensity. Representative fluorescent and bright-field images were selected at several angles to visualize a larger retinal field and converted to grayscale.

### Chord diagrams

Chord diagrams were generated to visualize behavioral data in the Barnes maze and the ViS4M (color and contrast modes) using the free resource Circos online at mkweb.bcgsc.ca/tableviewer/.

For the Barnes maze data, 20 × 20 data tables were created in Notepad according to the developer’s instructions. The segments of the chord diagrams correspond to the 20 holes of the Barnes maze and are color-coded by area on the platform from dark blue (closest holes to the escape box), light blue, green, yellow, orange, to red (hole on the opposite side of the escape box). The escape box is shown in black.

For ViS4M data, 4 × 4 data tables were created. The segments correspond to the four arms of the maze and are colored depending on the LED source (color mode) or the shade of the object (contrast mode). Ribbons represent the unidirectional transitions between two arms and are colored depending on the originating arm (segment). Chord diagrams show either the most frequent (upper Q4 quartile) or the least frequent transitions (lower Q1 quartile).

### Statistics

Data were analyzed using GraphPad Prism software, version 8 (GraphPad Software). Results are presented as individual data points and expressed as mean ± standard error of the mean (SEM). Two-group comparisons were analyzed using two-tailed unpaired Student’s t-tests. Three or more group comparisons were performed using a two-way ANOVA followed by *posthoc* Fisher’s LSD test for multiple comparisons. A *p* value of less than 0.05 was considered statistically significant. For linear regression, individual data points, fitted line, R-squared (R^2^) and *p* values are shown. The x-axis corresponds to the dependent variable (percentage of alternation) and the y-axis corresponds to the independent variables (predictors, percentage of transition).

### Ethical approval and guidelines

All experiments followed the NIH Guidelines for the Care and Use of Laboratory Animals and were approved by Cedars-Sinai Medical Center Institutional Animal Care and Use Committee. The study was carried out in compliance with the ARRIVE guidelines.

## Supplementary information

Supplementary Figures.

## Data Availability

Data are available from the authors upon reasonable request.
