# Supplementary material for: Color and contrast vision in mouse models of aging and Alzheimer’s disease using a novel visual-stimuli four-arm maze
Source: Sci Rep. 2021 Jan 13;11:1255. doi: 10.1038/s41598-021-80988-0 (PMC7806734; doi:10.1038/s41598-021-80988-0)
Supplement: Supplementary file 1 — Supplementary Figures. [file 41598_2021_80988_MOESM1_ESM.pdf]

Supplementary Data

Color and contrast vision in mouse models of aging and Alzheimer's disease using a novel visual-stimuli four-arm maze

Jean-Philippe Vit[1,2], Dieu-Trang Fuchs[3], Ariel Angel[4], Aharon (Ronnie) Levy[4], Itschak Lamensdorf[4], Keith L. Black[3], Yosef Koronyo[3], Maya Koronyo-Hamaoui[1,3]♣

[1]Department of Biomedical Sciences, Cedars-Sinai Medical Center, Los Angeles, CA, USA; [2]Biobehavioral Research Core, Cedars-Sinai Medical Center, Los Angeles, CA, USA; [3]Department of Neurosurgery, Maxine Dunitz Neurosurgical Research Institute, Cedars-Sinai Medical Center, Los Angeles, CA, USA; [4]Pharmaseed Ltd., 9 Hamazmera St., Ness Ziona 74047, Israel.

a

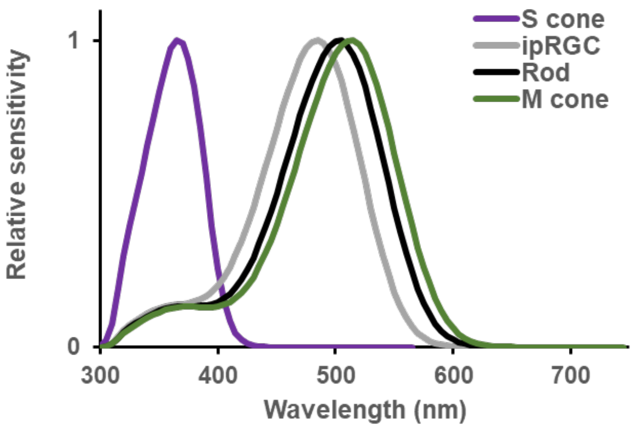

b

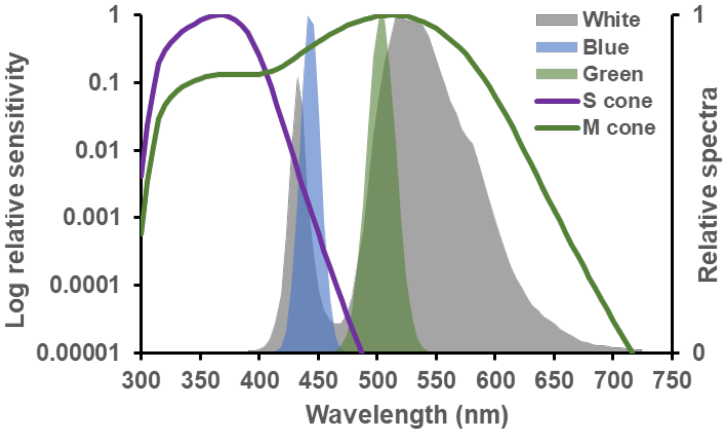

c

Low (L)	R	B	G	W
Illuminance	7	9	59	139
Luminance	0.3	0.4	2.9	7.0
Irradiance	4	29	14	34
Log photon flux	13.04	13.82	13.55	13.97
S-cone	3.21	10.78	7.63	10.74
Melanopsin	9.69	13.69	13.38	13.46
Rod	10.55	13.55	13.50	13.59
M-cone	10.99	13.46	13.53	13.65
Vision	M	M	M	M/P

Medium (M)	R	B	G	W
Illuminance	38	63	432	752
Luminance	1.9	3.0	20.4	40.1
Irradiance	19	205	100	182
Log photon flux	13.78	15.09	14.42	14.70
S-cone	3.95	11.63	8.50	11.47
Melanopsin	10.42	14.53	14.24	14.20
Rod	11.28	14.39	14.37	14.32
M-cone	11.73	14.30	14.39	14.38
Vision	M	M	P	P

High (H)	R	B	G	W
Illuminance	97	166	1040	2744
Luminance	4.9	8.3	52.0	137.2
Irradiance	49	540	241	664
Log photon flux	14.19	14.92	14.80	15.26
S-cone	4.35	12.05	8.88	12.04
Melanopsin	10.83	14.96	14.62	14.76
Rod	11.69	14.81	14.75	14.89
M-cone	12.13	14.72	14.77	14.94
Vision	M/P	M/P	P	P

Red High (RH)	R	B	G	W
Illuminance	103	9	66	164
Luminance	4.4	0.4	3.2	8.1
Irradiance	52	29	14	40
Log photon flux	14.21	13.82	13.60	14.04
S-cone	4.37	10.78	7.68	10.81
Melanopsin	10.86	13.69	13.42	13.53
Rod	11.71	13.55	13.55	13.66
M-cone	12.16	13.46	13.58	13.72
Vision	M/P	M	M/P	M/P

Equal (E)	R	B	G	W
Illuminance	76	75	73	67
Luminance	3.7	3.6	3.7	3.3
Irradiance	38	244	17	16
Log photon flux	14.08	14.74	13.64	13.65
S-cone	4.24	11.70	7.73	10.42
Melanopsin	10.72	14.61	13.47	13.15
Rod	11.58	14.47	13.59	13.27
M-cone	12.03	14.38	13.62	13.33
Vision	M/P	M/P	M/P	M/P

Illuminance: lux
Luminance: cd/m^2

Irradiance: μW/cm^2
Log photon flux: $\log_{10}$ photons/cm^2/s

S-cone, Melanopsin, Rod, M-cone:
$\log_{10}$ effective photons/cm^2/s

Vision: mesopic (M), photopic (P)

Supplementary Figure 1. Sensitivity of mouse opsins and specifications of light stimuli for all conditions

Supplementary Figure 1. Sensitivity of mouse opsins and specifications of light stimuli for all conditions. (**a**) Spectral sensitivity of mouse visual pigments. (**b**) Superposition of log relative sensitivity of cone opsins and relative spectra of blue, green and white LED lights. (**c**) Specifications of the four stimuli (red, blue, green, and white) for the five color-mode conditions (L, M, H, RH, and E) in photometric and radiometric units.

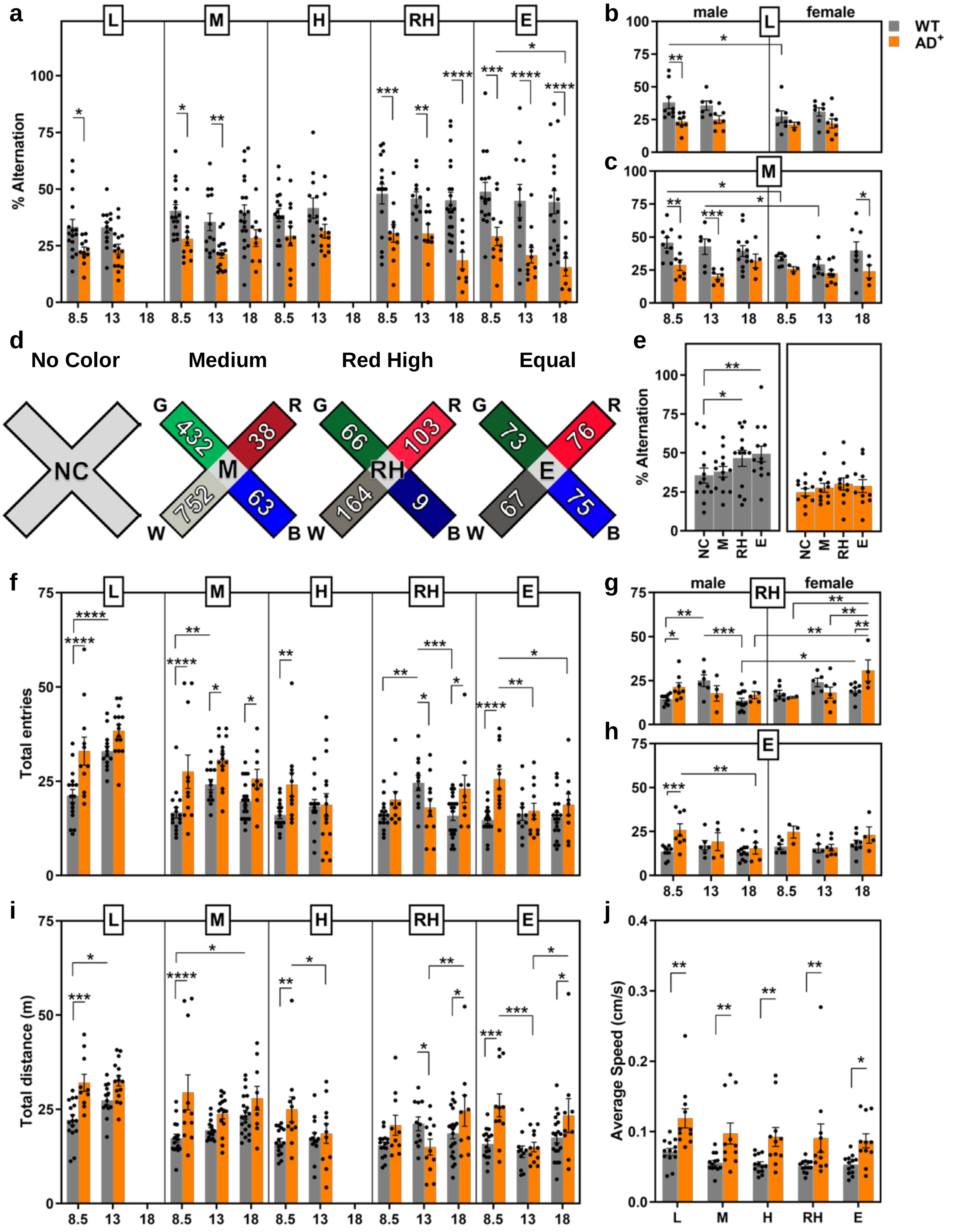

Supplementary Figure 2. Alternation, total entries, distance and speed of 8.5-, 13- and 18-month-old WT and AD⁺ mice

Supplementary Figure 2. Alternation, total entries, distance and speed of 8.5-, 13- and 18-month-old WT and AD^+ mice. (**a-c**) Percentage of alternation in 8.5-, 13- and 18-month-old WT and AD^+ mice under all five conditions (L, M, H, RH and E) of light intensity (**a**) and comparison by gender between WT and AD^+ mice under L (**b**) and M conditions (**c**). (**d**) Testing parameters under four light intensity conditions: No Color (NC), Medium (M), Red High (RH) and Equal (E). (**e**) Percentage of alternation in 8.5-month-old cohort under NC, M, RH and E conditions. (**f-h**) Total entries of 8.5-, 13- and 18-month-old WT and AD^+ mice under all five conditions of light intensity (**f**) and comparison by gender between WT and AD^+ mice under RH (**g**) and E conditions (**h**). (**i**) Total distance traveled of 8.5-, 13- and 18-month-old WT and AD^+ mice under all five conditions of light intensity. (**j**) Average speed of 8.5-month-old WT and AD^+ mice under all five conditions of light intensity. Mouse cohorts: 8.5-month-old WT (n=16; 9 males, 7 females) and AD^+ (n = 11; 8 males, 3 females); 13-month-old WT (n=11-15; 6-8 males, 5-7 females) and AD^+ (n = 11-15; 4-7 males, 7-8 females); 18-month-old WT (n=19; 11 males, 8 females) and AD^+ (n = 9; 5 males, 4 females). Group means, SEMs and individual data points are shown. * $p<0.05$, ** $p<0.01$, *** $p<0.001$, **** $p<0.0001$, by two-way ANOVA followed by *posthoc* Fisher's LSD test.

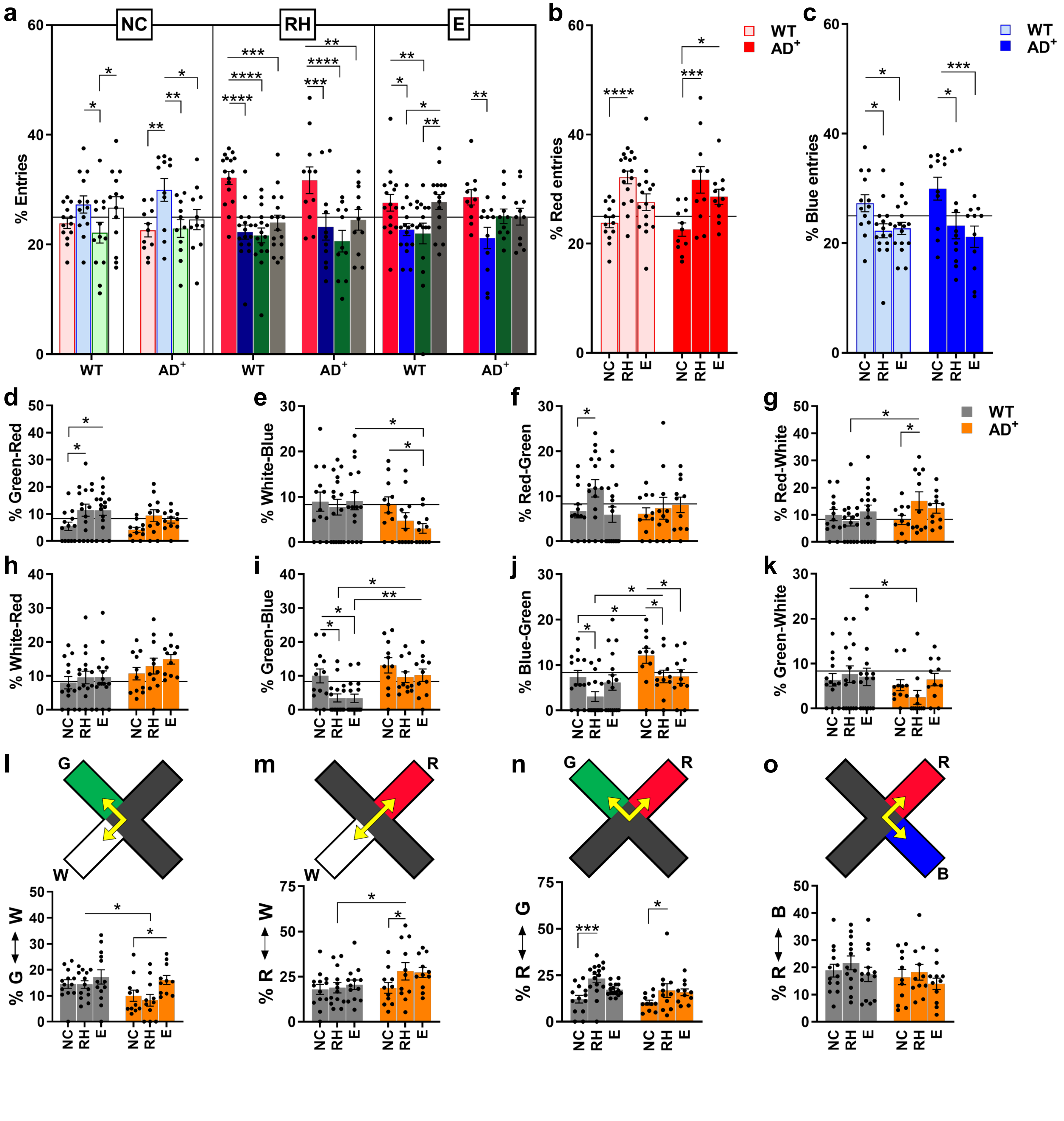

Supplementary Figure 3. Entries and transitions of 8.5-month-old WT and AD+ mice under control no-color condition

Supplementary Figure 3. Entries and transitions of 8.5-month-old WT and AD^+ mice under control no-color (NC) condition. (**a**) Percentage of entries under NC, RH and E conditions. (**b-c**) Comparison of the percentage of entries in red arm (**b**) and blue arm (**c**) between NC, RH and E conditions. (**d-k**) Percentage of unidirectional transitions between two colored arms as follows: green to red (**d**), white to blue (**e**), red to green (**f**), red to white (**g**), white to red (**h**), green to blue (**i**), blue to green (**j**), and green to white (**k**). (**l-o**) Percentage of bidirectional transitions under NC, RH and E conditions as shown in top panel and as follows: between green and white (**l**), blue and white (**m**), blue and green (**n**), and red and white arms (**o**). Mouse cohorts: 8.5-month-old WT (n=16) and AD^+ (n=11). Group means, SEMs and individual data points are shown. * $p<0.05$, ** $p<0.01$, *** $p<0.001$, **** $p<0.0001$, by two-way ANOVA followed by *posthoc* Fisher's LSD test.

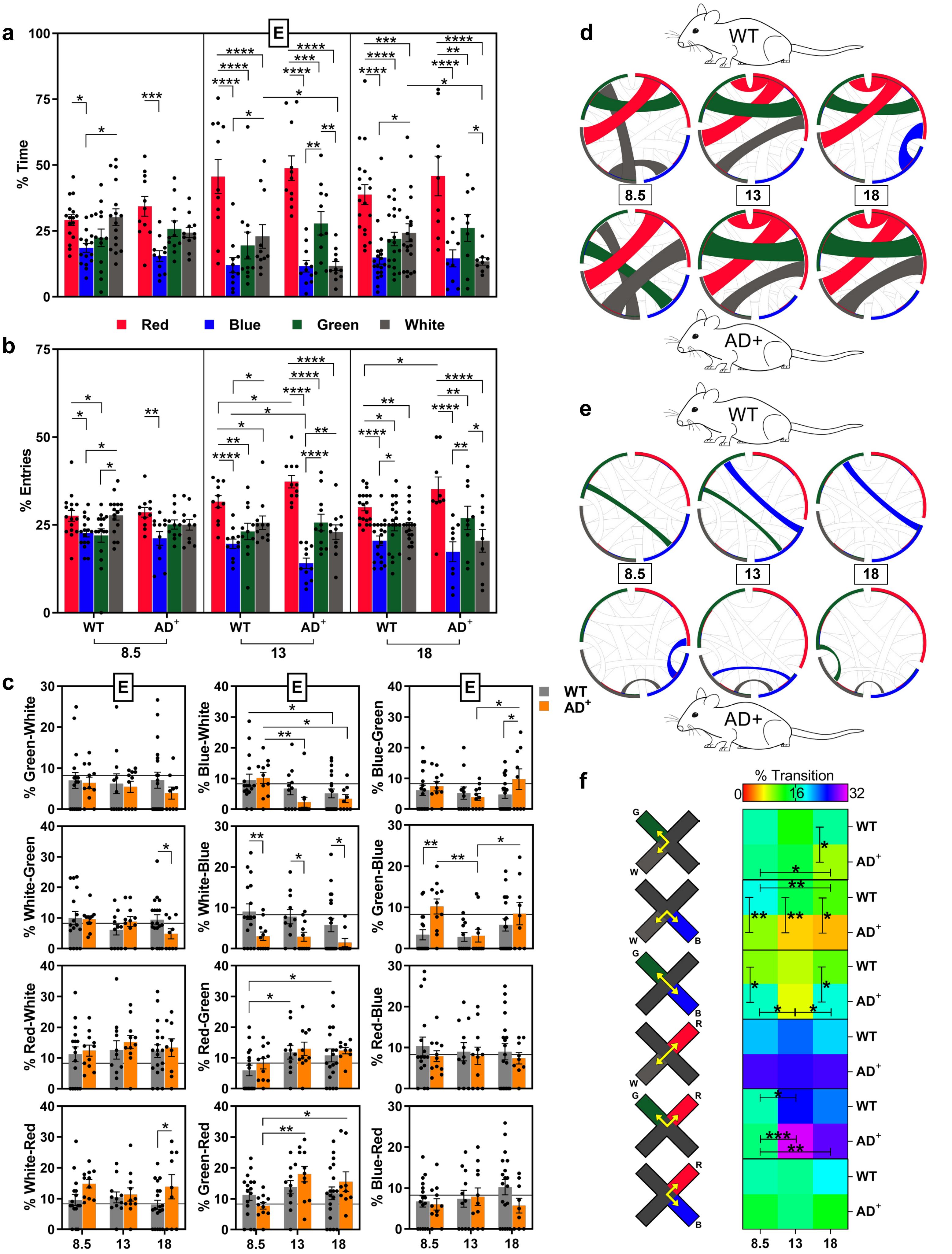

Supplementary Figure 4. Time, entries and transitions of 8.5-, 13- and 18-month-old WT and AD$^+$ mice under E condition

Supplementary Figure 4. Time, entries and transitions of 8.5-, 13- and 18-month-old WT and AD^+ mice under E condition. (**a**) Percentage of time spent in the different colored arms. (**b**) Percentage of entries. (**c**) Percentage of transitions between the different colored arms. (**d-e**) Chord diagrams depicting the most frequent transitions (**d**) and the least frequent transitions (**e**). (**f**) Rainbow heat map illustrating the percentage of the six types of bidirectional transitions. The top color gradient bar shows the range of percent transitions (from lowest in red to highest in purple). Mouse cohorts: 8.5-month-old WT (n=16) and AD^+ (n=11); 13-month-old WT (n=11-15) and AD^+ (n=11-15); 18-month-old WT (n=19) and AD^+ (n=9). Group means, SEMs and individual data points are shown. * $p<0.05$, ** $p<0.01$, *** $p<0.001$, **** $p<0.0001$, by two-way ANOVA followed by *posthoc* Fisher's LSD test.

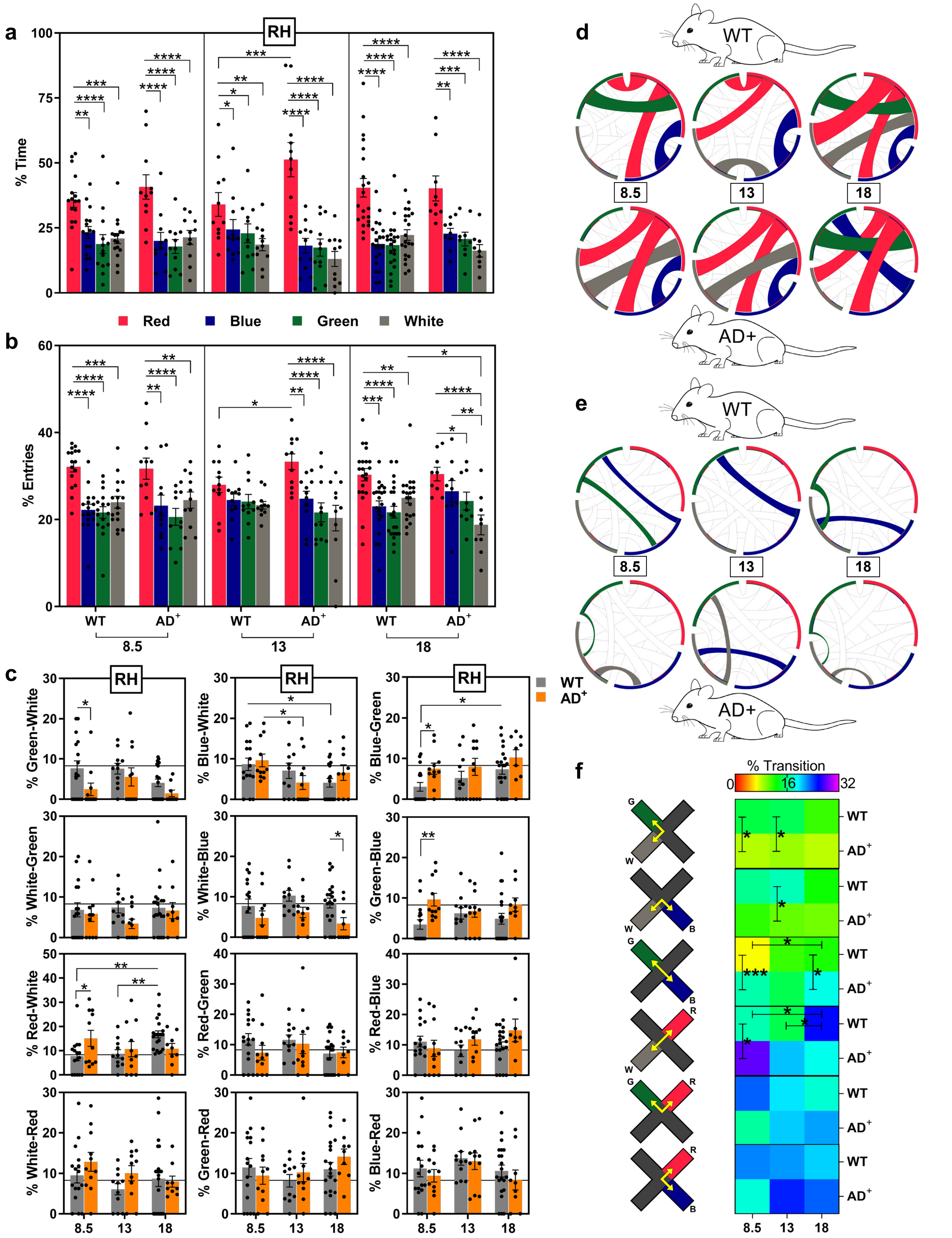

Supplementary Figure 5. Time, entries and transitions of 8.5-, 13- and 18-month-old WT and AD+ mice under RH condition

Supplementary Figure 5. Time, entries and transitions of 8.5-, 13- and 18-month-old WT and AD^+ mice under RH condition. (**a**) Percentage of time spent in the different colored arms. (**b**) Percentage of entries. (**c**) Percentage of transitions between the different colored arms. (**d-e**) Chord diagrams depicting the most frequent transitions (**d**) and the least frequent transitions (**e**). (**f**) Rainbow heat map illustrating the percentage of the six types of bidirectional transitions. The top color gradient bar shows the range of percent transitions (from lowest in red to highest in purple). Mouse cohorts: 8.5-month-old WT (n=16) and AD^+ (n=11); 13-month-old WT (n=11-15) and AD^+ (n=11-15); 18-month-old WT (n=19) and AD^+ (n=9). Group means, SEMs and individual data points are shown. * $p<0.05$, ** $p<0.01$, *** $p<0.001$, **** $p<0.0001$, by two-way ANOVA followed by *posthoc* Fisher's LSD test.

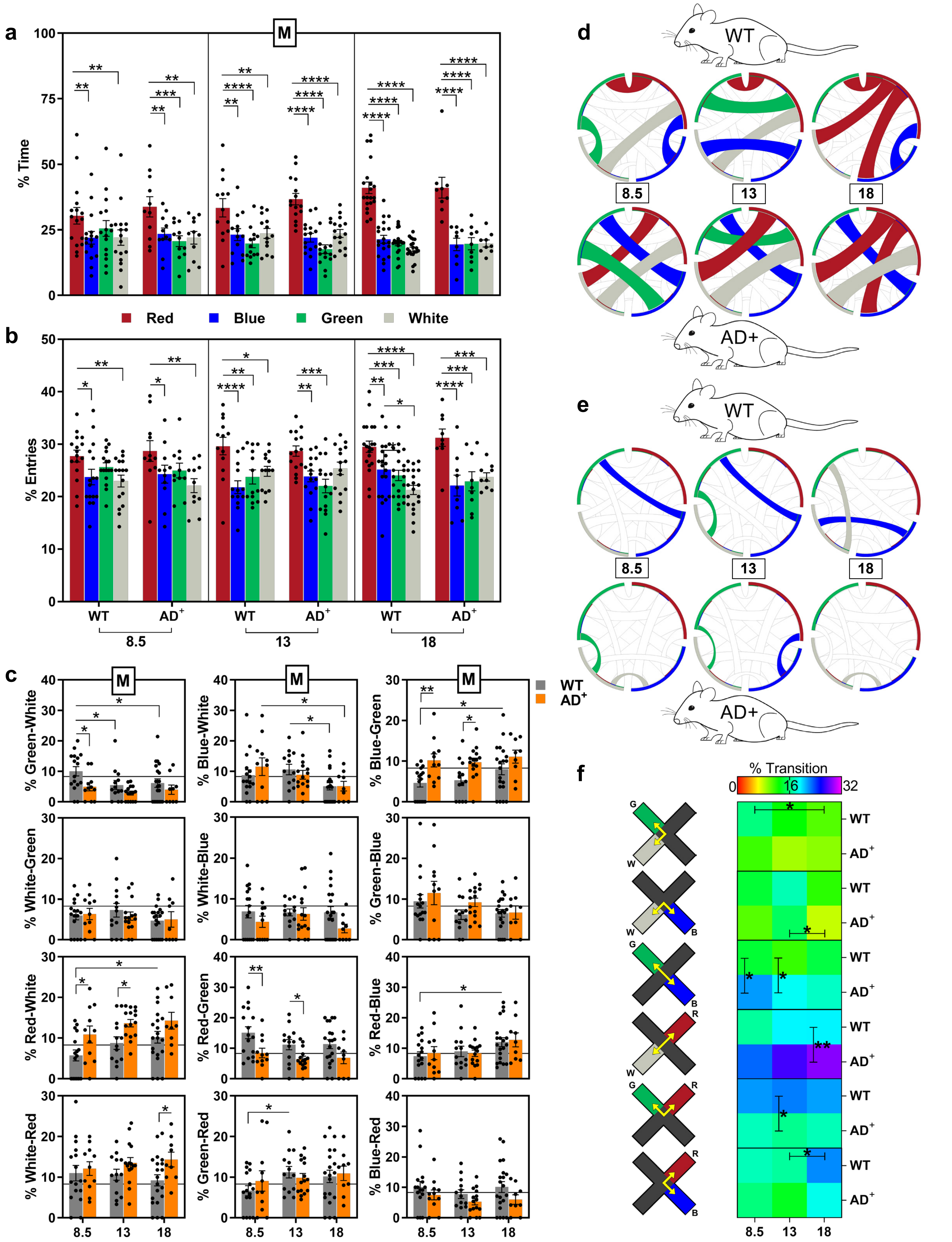

Supplementary Figure 6. Time, entries and transitions of 8.5-, 13- and 18-month-old WT and AD+ mice under M condition

Supplementary Figure 6. Time, entries and transitions of 8.5-, 13- and 18-month-old WT and AD^+ mice under M condition. (**a**) Percentage of time spent in the different colored arms. (**b**) Percentage of entries. (**c**) Percentage of transitions between the different colored arms. (**d-e**) Chord diagrams depicting the most frequent transitions (**d**) and the least frequent transitions (**e**). (**f**) Rainbow heat map illustrating the percentage of the six types of bidirectional transitions. The top color gradient bar shows the range of percent transitions (from lowest in red to highest in purple). Mouse cohorts: 8.5-month-old WT (n=16) and AD^+ (n=11); 13-month-old WT (n=11-15) and AD^+ (n=11-15); 18-month-old WT (n=19) and AD^+ (n=9). Group means, SEMs and individual data points are shown. * $p<0.05$, ** $p<0.01$, *** $p<0.001$, **** $p<0.0001$, by two-way ANOVA followed by *posthoc* Fisher's LSD test.

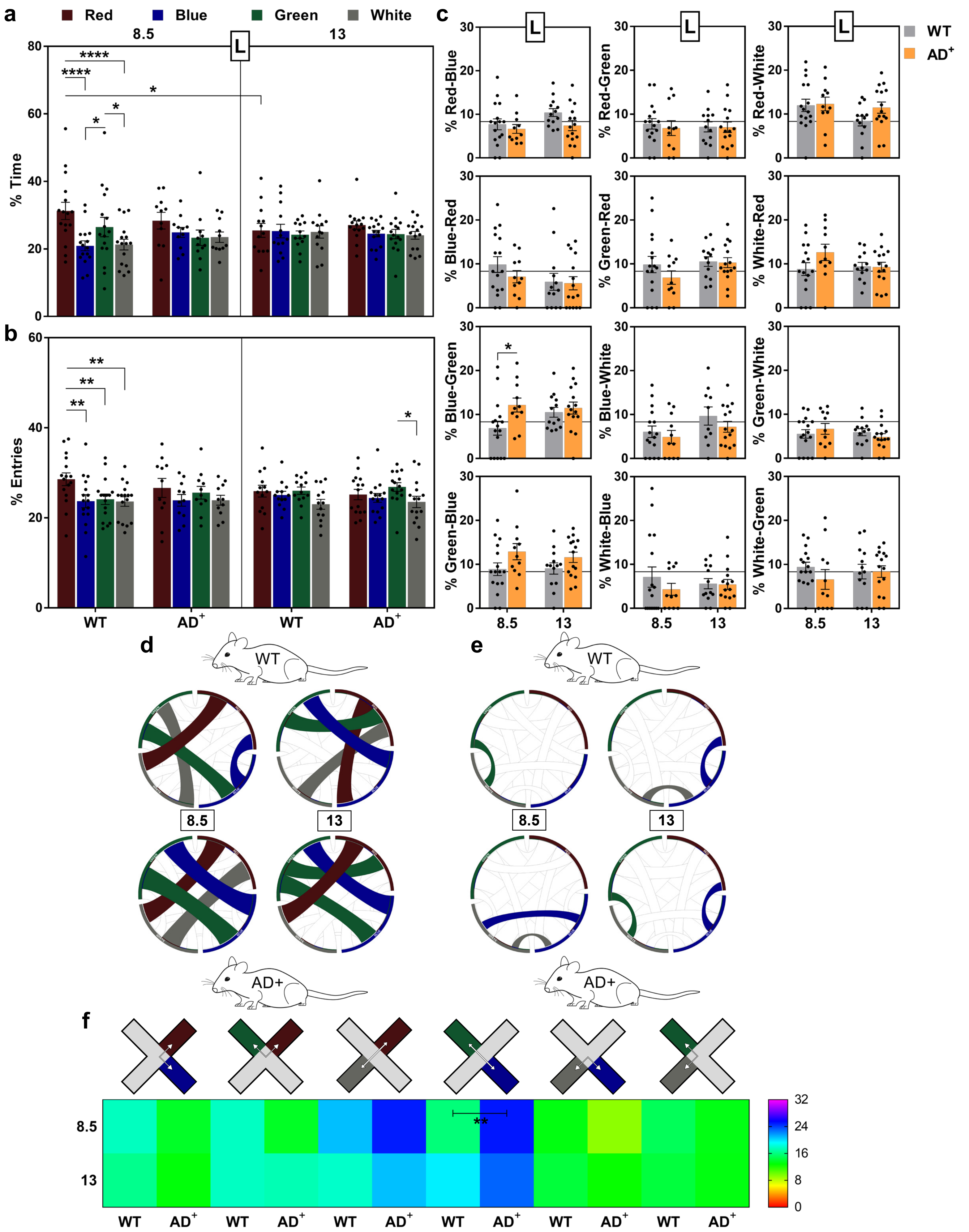

Supplementary Figure 7. Time, entries and transitions of 8.5- and 13-month-old WT and AD^+ mice under L condition

Supplementary Figure 7. Time, entries and transitions of 8.5- and 13-month-old WT and AD^+ mice under L condition. (**a**) Percentage of time spent in the different colored arms. (**b**) Percentage of entries. (**c**) Percentage of transitions between the different colored arms. (**d-e**) Chord diagrams depicting the most frequent transitions (**d**) and the least frequent transitions (**e**). (**f**) Rainbow heat map illustrating the percentage of the six types of bidirectional transitions. The color gradient bar on the right shows the range of percent transitions (from lowest in red to highest in purple). Mouse cohorts: 8.5-month-old WT (n=16) and AD^+ (n=11); 13-month-old WT (n=11-15) and AD^+ (n=11-15). Group means, SEMs and individual data points are shown. * $p<0.05$, ** $p<0.01$, *** $p<0.001$, **** $p<0.0001$, by two-way ANOVA followed by *posthoc* Fisher's LSD test.

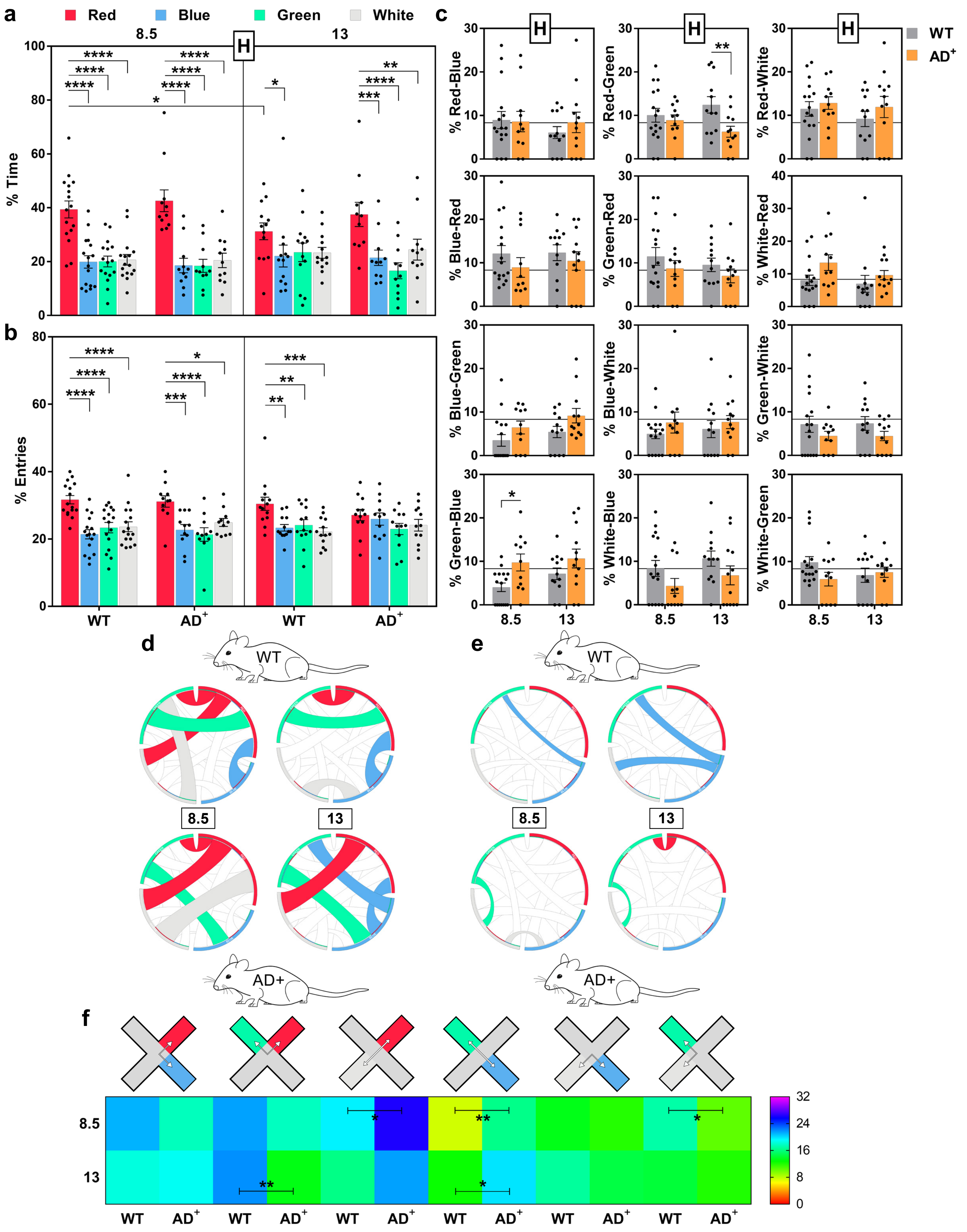

Supplementary Figure 8. Time, entries and transitions of 8.5- and 13-month-old WT and AD⁺ mice under H condition

Supplementary Figure 8. Time, entries and transitions of 8.5- and 13-month-old WT and AD^+ mice under H condition. (**a**) Percentage of time spent in the different colored arms. (**b**) Percentage of entries. (**c**) Percentage of transitions between the different colored arms. (**d-e**) Chord diagrams depicting the most frequent transitions (**d**) and the least frequent transitions (**e**). (**f**) Rainbow heat map illustrating the percentage of the six types of bidirectional transitions. The color gradient bar on the right shows the range of percent transitions (from lowest in red to highest in purple). Mouse cohorts: 8.5-month-old WT (n=16) and AD^+ (n=11); 13-month-old WT (n=11-15) and AD^+ (n=11-15). Group means, SEMs and individual data points are shown. * $p<0.05$, ** $p<0.01$, *** $p<0.001$, **** $p<0.0001$, by two-way ANOVA followed by *posthoc* Fisher's LSD test.

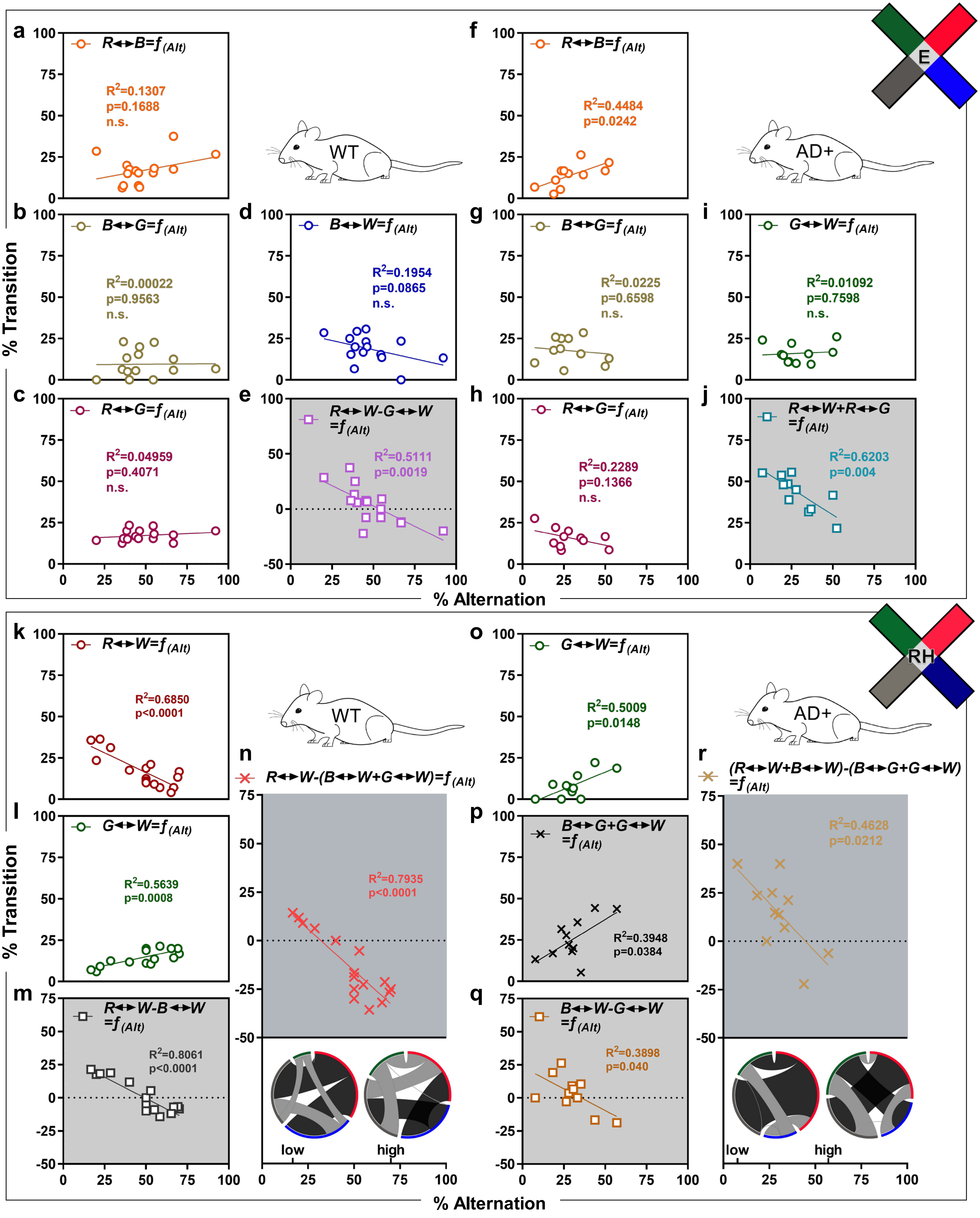

Supplementary Figure 9. Correlation between alternation and color transitions in 8.5-month-old WT and AD⁺ mice

Supplementary Figure 9. Correlation between alternation and color transitions in 8.5-month-old WT and AD^+ mice. (**a-j**) Significant and non-significant linear regressions under E condition in WT (**a-e**) and AD^+ mice (**f-j**) of the percentage of alternation with the percentage of single bidirectional transitions (**a-d, f-i**); or the percentage of negatively paired bidirectional transitions (**e**); or the percentage of positively paired bidirectional transitions (**j**). (**k-r**) Most significant linear regressions under RH condition in WT (**k-n**) and AD^+ mice (**o-r**) of the percentage of alternation with the percentage of single bidirectional transitions (**k, l, o**); or the percentage of negatively paired bidirectional transitions (**m, q**); or the percentage of positively paired bidirectional transitions (**p**); or the percentage of combined positively and negatively paired bidirectional transitions (**n, r**). (**n, r**) Inserts are chord diagrams depicting bidirectional transitions of the animals with the lowest (low) and highest (high) percentage of alternation. Mouse cohorts: 8.5-month-old WT (n=16) and AD^+ (n=11). In all graphs, individual data points, fitted line, R-squared (R^2) and p-values are shown. The x-axis corresponds to the dependent variable (percentage of alternation) and the y-axis corresponds to the independent variables (predictors, percentage of transition).
